# Clinical, lifestyle, environmental and dietary determinants of malnutrition in adolescents on antiretroviral therapy in Ethiopia

**DOI:** 10.1371/journal.pgph.0005003

**Published:** 2026-06-26

**Authors:** Meless Gebrie Bore, Lin Perry, Xiaoyue Xu, Andargachew Kassa Biratu, Marilyn Cruickshank

**Affiliations:** 1 College of Medicine and Health Sciences, Hawassa University, Hawassa, Ethiopia; 2 School of Nursing and Midwifery, Faculty of Health, University of Technology Sydney, Ultimo, New South Wales, Australia; 3 School of Population Health, Faculty of Medicine, University of New South Wales, Kensington, New South Wales, Australia; 4 Sydney Children’s Hospitals Network, Sydney, Australia; PLOS: Public Library of Science, UNITED STATES OF AMERICA

## Abstract

Adolescence is a critical period for growth and development, and adequate nutrition is essential for adolescents living with HIV (ALHIV) receiving antiretroviral therapy (ART). This study examined factors associated with malnutrition among ALHIV receiving ART in Ethiopia. A facility-based cross-sectional study included 384 ALHIV aged 10–19 years attending ten public hospitals in Addis Ababa and Oromia from August to December 2023. Data were collected using a structured questionnaire, clinical assessments, and record reviews. Multivariable logistic regression identified factors associated with malnutrition. Overall, 24.2% of participants were thin, 21.7% were stunted, and 34.9% were acutely malnourished. Females had lower odds of thinness (AOR = 0.27; 95% CI: 0.16–0.45). Absence of chronic infections (AOR = 0.48; 95% CI: 0.29–0.80), absence of anxiety symptoms (AOR = 0.53; 95% CI: 0.32–0.88), and nutritional supplementation (AOR = 0.55; 95% CI: 0.32–0.95) were associated with lower odds of thinness, whereas food insecurity increased the odds (AOR = 1.63; 95% CI: 1.02–2.62). Acute malnutrition was associated with younger age (10–17 years) (AOR = 1.94; 95% CI: 1.03–3.64), larger household size (AOR = 2.27; 95% CI: 1.23–4.17), shorter duration of HIV status awareness (AOR = 2.96; 95% CI: 1.62–5.40), low haemoglobin levels (AOR = 7.29; 95% CI: 1.31–40.5), food insecurity (AOR = 1.85; 95% CI: 1.09–2.14), and low meal frequency (AOR = 1.86; 95% CI: 1.08–3.19) were associated with increased odds of acute malnutrition. Females (AOR = 0.58; 95% CI: 0.38–0.88), absence of anxiety symptoms (AOR = 0.50; 95% CI: 0.28–0.89), and nutritional supplementation were protective against acute malnutrition (AOR = 0.45; 95% CI: 0.36–0.79). Malnutrition among ALHIV receiving ART in Ethiopia is influenced by clinical, socioeconomic, and dietary factors. Integrated nutrition, clinical care, and food security interventions are needed to improve outcomes.

## 1. Introduction

Adolescence is defined by the World Health Organization (WHO) as the period between 10 and 19 years of age and is a period marked by rapid physical, emotional and social development, making adequate nutritional status essential for this age group [1,2]. For adolescents living with HIV/AIDS, maintaining adequate nutrition is vital not only for overall health but also for the effectiveness of antiretroviral therapy (ART) [3]. Globally, HIV/AIDS remains a significant public health challenge, with approximately 39 million people living with HIV worldwide, including an estimated 1.7 million adolescents aged 10–19 years who are affected by HIV [4]. Adolescents are uniquely vulnerable due to a combination of developmental, social, and economic factors, including limited access to healthcare services, stigma, and increased engagement in high-risk behaviours [5,6]. In Ethiopia, despite a decline in overall HIV prevalence—from 2.3% in 2002 to 0.8% in 2021 [7]—adolescents continue to experience a substantial burden of HIV. For example, recent data indicate that adolescents account for a growing proportion of new HIV infections and face unique challenges in accessing care and support [6,8]. This vulnerability is compounded by limited access to healthcare, pervasive stigma and engagement in high-risk behaviours [8].

Adolescents have distinct nutritional needs and require increased energy, protein, vitamins, and minerals to support their growth and development [9]. However, many adolescents on ART face unique challenges, including stigma, dietary restrictions, and comorbidities, which may negatively affect nutritional intake [10]. Socioeconomic factors, cultural practices, and difficulties accessing healthcare services are also associated with poor nutritional status in this population [11]. Inadequate nutritional status among these adolescents can impair immune function and reduce the effectiveness of therapy, increasing morbidity and mortality [12,13].

In Ethiopia, malnutrition among adolescents remains a major public health concern. National and regional studies have reported substantial levels of thinness, stunting, and micronutrient deficiencies among adolescents, particularly among those affected by chronic illnesses and socio-economic disadvantage [11,14]. Adolescents living with HIV are especially vulnerable due to increased metabolic demands, recurrent infections, treatment-related complications, food insecurity, and psychosocial challenges [3,15]. Evidence from sub-Saharan Africa indicates that adolescents receiving ART frequently experience comorbidities, mental health problems, stigma, and episodes of virological non-suppression, all of which may negatively affect nutritional status, treatment adherence, and overall health outcomes [4,6,8,16]. Studies have also reported virological non-suppression rates ranging from approximately 20% to 28% among adolescents on ART [17], while depression and anxiety remain highly prevalent in this population [8,15,16].

Despite improvements in HIV care and ART coverage, these interconnected challenges continue to affect the well-being of adolescents living with HIV in Ethiopia. Nevertheless, significant gaps persist despite the Ethiopian Ministry of Health’s efforts to develop and implement adolescent and youth health programs [18]. While studies have emphasized the need for tailored nutritional interventions for individuals on ART [19], comprehensive research focusing specifically on adolescents in developing countries, including Ethiopia, remains limited, particularly studies that integrate clinical, lifestyle, environmental and dietary determinants of nutritional status. This study aimed to examine these factors among adolescents on ART recruited as outpatients from selected Ethiopian hospitals. Findings will contribute to the current body of knowledge to inform policymakers and healthcare providers about the specific nutritional needs and challenges faced by adolescents on ART, contributing to strategies that optimize ART effectiveness and promote adolescent well-being.

## 2. Methods

### 2.1. Ethics statements

This study was conducted in accordance with the Declaration of Helsinki. Ethical approval was obtained from the Human Research Ethics Committee, University of Technology Sydney, Australia (Ref: ETH23-7873); the Institutional Review Board of the College of Medicine and Health Sciences, Hawassa University, Ethiopia (Ref: IRB/321/15); the Regional Health Bureau Ethics Review Committee; and the Ethics Committees of the participating hospitals. Permission was secured from all health institutions. Confidentiality was maintained throughout the study.

Written informed consent was obtained from all participants aged 18–19 years prior to data collection. For participants younger than 18 years, written parental or guardian consent and adolescent assent were obtained before participation. Participants were informed about the purpose of the study, confidentiality of information, voluntary participation, and their right to withdraw from the study at any time without affecting their clinical care.

### 2.2. Study setting and period

This research was conducted in selected public health facilities providing ART for adolescents living with HIV (ALHIV) in Ethiopia. The study was carried out from September 2023 to February 2024 at 10 large public referral and general hospitals located in urban and peri-urban areas of Addis Ababa and the Oromia region. Addis Ababa has one of the highest HIV prevalence rates in Ethiopia, with adult HIV prevalence estimates of approximately 3.4%, while Oromia has a substantial number of people receiving HIV care and ART services despite relatively lower regional HIV prevalence rates. HIV transmission in Ethiopia remains predominantly heterosexual, with ongoing new infections reported among adolescents and young people, particularly in urban settings [20]. The selected hospitals were purposively chosen based on high HIV case burden, high adolescent ART clinic enrolment, and the availability of comprehensive HIV care and follow-up services. These hospitals provide outpatient HIV care, ART follow-up, nutritional assessment, counselling, viral load monitoring, and psychosocial support services for adolescents and their families. All participants were recruited from outpatient ART clinics during routine follow-up visits; no inpatients were included in the study.

### 2.3. Study design

An institution-based mixed-methods design was employed. The predominant quantitative component used a cross-sectional survey design, supplemented by clinical assessments and extraction of clinical record data. The survey consisted of a composite structured, interviewer-administered questionnaire. A small qualitative component used open-ended questions to provide deeper insights. The study used a mixed-methods sequential explanatory design, in which quantitative data were collected and analysed first, followed by qualitative responses used to contextualize, explain, and enrich the quantitative findings. Integration of quantitative and qualitative findings occurred during interpretation of the results to provide a more comprehensive understanding of factors influencing nutritional status among adolescents living with HIV. Integrating quantitative and qualitative data enabled a holistic understanding of factors, barriers, and facilitators to optimal nutrition for ALHIV. Integrating quantitative and qualitative data enabled a holistic understanding of factors, barriers and facilitators to optimal nutrition for ALHIV.

Study reportage adhered to the Strengthening the Reporting of Observational Studies in Epidemiology-Nutritional Epidemiology (STROBE-Nut) guidelines for observational nutritional research [21–23]. Operational definitions are provided in S1 Text).

### 2.4. Selection criteria and the sampling process

Adolescent living with HIV aged 10–19 years who were on ART and attending ART units in the selected hospitals were eligible for inclusion.

Adolescents were excluded if they had been on ART for less than 3 months because this early treatment period may be insufficient for clinical and nutritional stabilization and may reflect temporary physiological and nutritional changes associated with ART initiation. Excluding these participants helped ensure a more stable assessment of nutritional status and treatment-related factors among adolescents established on ART. Adolescents were also excluded if they had cognitive or communication deficits, were younger than 18 years without parental or guardian consent, lacked a verifiable registration number, or were admitted as inpatients.

**Sample size determination**: Sample size was calculated to address the study objectives related to nutritional status, associated factors, and dietary practices among adolescents living with HIV receiving ART. For estimation of the prevalence of malnutrition, the sample size was determined using the single population proportion formula with a 95% confidence level, 5% margin of error, and an estimated prevalence of undernutrition of 33.1% obtained from a previous study conducted among adolescents living with HIV in southern Ethiopia [14].


$$\begin{array}{c} n=\frac{\left(Z_{\alpha /\text{2}}{)}^{\text{2}}P(\text{1}-P\right)}{d^{\text{2}}} \end{array}$$

where *n* = required sample size, $Z_{\alpha /\text{2}}$ = 1.96 at 95% confidence level, *P* = estimated prevalence of undernutrition (33.1%), and *d* = margin of error (0.05). The calculated sample size for prevalence estimation was 340.

Sample size for factors associated with malnutrition was additionally estimated using a two-population proportion formula based on predictors identified in previous studies, assuming 95% confidence level, 80% power, and 1:1 ratio of exposed to unexposed groups [14,24]. The largest calculated sample size for these analyses was 352. For dietary assessment objectives, a prevalence estimate of 50% was used due to limited prior evidence, yielding a required sample size of 384 participants. The largest calculated sample size (384) was adopted for the study.

A proportionate random sampling approach was used. Eligible participants were identified using ART registration codes, assigned unique research identifier, and randomly selected within each hospital using SPSS version 26.

The final sample size was proportionally allocated across participating hospitals based on the number of adolescents enrolled in ART follow-up care at each facility using probability proportional to size (PPS) allocation (see S2 Text).

### 2.5. Data collection

Data were collected using a pre-tested, interviewer-administered composite structured questionnaire, clinical assessments, and clinical record review. The questionnaire gathered quantitative data, including sociodemographic, clinical, lifestyle, environmental, and dietary factors related to nutritional status of ALHIV on ART. Open-ended questions were included to capture personal experiences and contextual factors related to nutrition. The same participants contributed to both the quantitative survey and qualitative open-ended responses. Clinical assessments and data clinical record reviews provided objective health and nutritional measurements.

Healthcare professionals, including nurses, health officers, public health practitioners, and nutritionists, conducted the interviews using smartphones and computers, with data entered using the Kobo Toolbox program. The healthcare professionals who collected the study data were not directly involved in the routine clinical care or service provision of the participating adolescents. This approach was used to minimize potential response and social desirability bias during data collection. A three-day training session was conducted prior to data collection, covering the study’s objectives, interview techniques, and standardized procedures for anthropometric measurements, clinical and dietary assessments.

The research team supervised the data collection process to ensure adherence to protocol. In case of technical issues with the platform, printed questionnaires were available. Daily checking of data completeness and accuracy, and data cleaning ensured quality control and data integrity. Consistency among raters was assessed using Cohen’s Kappa coefficient, with scores of ≥ 0.71 indicating substantial agreement [25].

#### 2.5.1. Components of the questionnaire.

The composite structured questionnaire was adapted from previously validated instruments and published literature to align with the study objectives and local context. It included both closed-ended and open-ended questions addressing sociodemographic characteristics, anthropometric, clinical (HIV and mental health), lifestyle, environmental, and dietary variables. Standardized and validated tools included the Generalized Anxiety Disorder Questionnaire (GAD-7) [26], Patient Health Questionnaire-9 (PHQ-9) [27], Alcohol Use Disorders Identification Test-Consumption (AUDIT-C) [28,29], Household Food Insecurity Access Scale (HFIAS) [30], and Individual Dietary Diversity Score (IDDS) [31].

The sociodemographic characteristics collected included age, sex, education level, religion, household composition, household income, school attendance, and recruitment site.

Anthropometric measurements were collected to assess nutritional status, following standardized protocols to ensure accuracy and consistency. Weight was measured with Seca digital weighing scales (SECA GmbH, Hamburg, Germany), and height was recorded using portable stadiometers to calculate BMI as kg/m^2^. Circumferences (upper arm, waist, and hip) were assessed using a Holtain measuring tape, while skinfold thickness was measured at standardized sites (i.e., triceps, biceps, subscapular, and supra-iliac) with Harpenden callipers to estimate body fat percentage. Body composition was evaluated using a handheld bioelectrical impedance analyser (BIA; Tanita BC-558) [32,33]. Hand grip strength was assessed with a dynamometer (Jamar Hydraulic Hand Dynamometer, Model 5030J1) [34], recording the highest value from three squeezes of both hands. Reliability of anthropometric measurements was confirmed using the Intraclass Correlation Coefficient (ICC = 0.856), indicating good agreement.

Physical assessments identified signs of malnutrition such as wasting, stunting, bilateral pitting oedema, recent weight loss, dermatosis, palmar pallor, and eye signs (e.g., Bitot spots, corneal cloudiness, and corneal ulceration) [33]. Clinical signs were assessed according to WHO nutritional assessment guidelines. Wasting was defined as visible loss of muscle mass and subcutaneous fat, bilateral pitting oedema as abnormal fluid accumulation associated with severe acute malnutrition, palmar pallor as clinical evidence suggestive of anaemia, dermatosis as skin change associated with nutritional deficiencies, and ocular signs such as Bitot spots, corneal cloudiness, and corneal ulceration as indicators of vitamin A deficiency and severe nutritional impairment [35].

Regular monitoring and timely evaluation of treatment adherence, immune recovery, and nutritional status are recognised as important for improving overall health outcomes among people living with HIV. Clinical record data extracted from patient health records included HIV status, haematological and biochemical markers, CD4 counts, viral load measurement, comorbidities, and nutritional deficiencies. The World Health Organization (WHO) and the Centres for Disease Control and Prevention (CDC) [36,37] recognize viral load assessment as a critical component of HIV management. Clinical records included baseline and follow-up HIV monitoring indicators, including CD4 counts and viral load measurements, which are routinely used in ART monitoring and evaluation to assess treatment response and overall clinical status [38].

Mental health (anxiety and depression) was assessed using the validated Generalized Anxiety Disorder Questionnaire (GAD-7; Cronbach’s α = 0.77) and Patient Health Questionnaire-9 (PHQ-9; Cronbach’s α = 0.85) [26,27]. Lifestyle factors included alcohol consumption, evaluated with AUDIT-C screening tool [29,39], which demonstrated high reliability with Cronbach’s α 0.847 in this study, consistent with previous research [40].

Environmental and dietary factors included household food security, access to clean water and sanitation, meal frequency, dietary diversity, and nutritional supplementation (e.g., RUTF). Household food security was evaluated using the Household Food Insecurity Access Scale (HFIAS) [30], and dietary diversity was measured using the Individual Dietary Diversity Score (IDDS) [31]. Open-ended questions drafted by the research team explored personal experiences and contextual factors that shaped food choices and eating practices among ALHIV.

#### 2.5.2. Questionnaire rigour.

The questionnaire was originally written in English, translated into Amharic and then back-translated by independent language experts for comparison [41]. Both versions were reviewed by local ART unit staff, including senior clinicians not employed at the study sites, to assess content validity and relevance, and appropriateness of language for the target population. The Item-Level Content Validity Index (I-CVI) and the Scale-Level Content Validity Index (S-CVI) were calculated [42,43], with a threshold of 0.78 set for acceptable content relevance [44]. Six experts—two from academia, two from clinical practice, and two from a regional health office—rated item relevance and clarity on a four-point scale. The mean S-CVI was 0.981, indicating strong content validity, with 98.1% of experts rating the items as moderately or highly relevant and clear. Qualitative feedback from experts was incorporated to refine wording and enhance clarity of the final questionnaire.

### 2.6. Data processing and analysis

All statistical analyses were conducted using SPSS version 26. Descriptive statistics summarized the sociodemographic, clinical, lifestyle, environmental and dietary characteristics of these adolescents on ART. Frequency tables and figures illustrated the distribution of variables to ensure data accuracy and identify any potential outliers or inconsistencies. Continuous variables were tested for normality using the Kolmogorov-Smirnov and Shapiro-Wilk tests (p < 0.05), further confirmed through Q-Q plots. Cases with missing values or incomplete data were excluded from the analysis to ensure consistency and maintain data integrity; the final sample size reflected complete responses only.

Binary outcome variables were created for regression analyses, including thinness (BMI-for-age Z-score <−2 SD versus ≥ −2 SD) and acute malnutrition based on age-specific MUAC cut-offs (malnourished versus not malnourished). These dichotomized outcome variables were analysed using binary logistic regression to identify factors associated with malnutrition among adolescents living with HIV receiving ART. Variables with p-values < 0.25 in the bivariate analysis were entered into multivariable logistic regression models using backward elimination, with statistical significance set at p < 0.05.

Multicollinearity was checked using the Variance Inflation Factor (VIF) and tolerance values; variables with VIF > 10 or tolerance < 0.1 were considered to indicate multicollinearity and were excluded from the final model. Interaction effects (e.g., gender × age) were examined to assess model robustness, and all other model assumptions were satisfied.

Qualitative data were initially categorized to facilitate integration with the quantitative dataset and complement statistical findings [45–47]. Qualitative responses were analysed manually using reflexive thematic analysis without the use of specialised qualitative data analysis software, as the qualitative component consisted of brief open-ended responses intended to contextualize the quantitative findings. Additionally, qualitative data were analysed using reflexive thematic analysis informed by Braun and Clarke’s approach [47], in which two independent researchers inductively coded qualitative responses to identify key themes. Themes were developed iteratively through repeated review, discussion, and consensus among the research team. Discrepancies were resolved by consensus, and representative illustrative quotes were selected to enrich the interpretation of findings. Each participant and healthcare provider were assigned an identifier to prevent duplication and ensure full de-identification. All analytical procedures adhered to the ethical standards and approvals outlined in the declarations section.

## 3. Results

### 3.1. Participants’ characteristics

A total of 384 adolescents living with HIV (ALHIV) attending ART follow-up at 10 hospitals in Addis Ababa and Oromia were enrolled, resulting in a 100% response rate.

#### 3.1.1. Socio-demographic characteristics.

The mean age of all participants was 15.9 (SD = 2.19) years and 53.9% (n = 207) were female. The largest group (59.1%, n = 227) were aged 14–17 years, representing mid-adolescence. The majority were Orthodox Christians (73%, n = 281), and 66.1% (n = 254) were recruited from Addis Ababa hospitals. Nearly all participants were students (98.7%, n = 379); 46.6% (n = 179) were attending grades 1–8. Regarding household income, the largest proportion reported a monthly income of 1000–3000 EBR (45.6%, n = 175). More than half of the participants lived in households with 4–5 members (51.6%, n = 198), and 96.1% (n = 369) lived with parents or a responsible adult (Table 1).

**Table 1:** Socio-demographic characteristics of ALHIV attending ART follow-up in Ethiopia, 2024 (n = 384).

Variables	Description	Frequency n (%)
Age (years)	10–13 years	55 (14.3%)
	14–17 years	227 (59.1%)
	18–19 years	102 (26.6%)
	Mean age ± SD	15.9 ± 2.19 years
Sex	Male	177 (46.1%)
	Female	207 (53.9%)
Region	Addis Ababa	254 (66.1%)
	Oromia	130 (33.9%)
Religion	Orthodox	281 (73.2%)
	Muslim	49 (12.8%)
	Protestant	48 (12.5%)
	Others (Catholic, Jehovah’s Witness)	6 (1.5%)
Highest educational grade	Grade 1–8	179 (46.6%)
	Grade 9–10	111 (28.9%)
	Grade 11 and above	94 (24.5%)
Occupation	Student	379 (98.7%)
	Daily labourer	3 (0.8%)
	Others (industry employees, begging)	2 (0.5%)
Family monthly income	<1000 EBR	41 (10.7%)
	1000– < 3000 EBR	175 (45.6%)
	≥3000–5000 EBR	94 (24.5%)
	≥ 5000 EBR	74 (19.3%)
Family size	< 4	130 (33.9%)
	4 – 5	198 (51.6%)
	≥ 6	56 (14.6%)
Living situation	Living with parents/responsible adult	369 (96.1%)
	Living alone or with peers	15 (3.9%)

SD = Standard Deviation; EBR = Ethiopian Birr.

#### 3.1.2. Anthropometric measures of nutritional status.

The nutritional status of participants was assessed using three measures: Body Mass Index-for-Age Z-scores (BAZ), Height-for-Age Z-scores (HFA), and Mid-Upper Arm Circumference (MUAC) (S1 Text).

Regarding thinness, 24.2% (n = 93) of participants had BMI-for-age Z-scores below -2 SD. Of these, 26.9% (n = 25) were classified as severely thin (Z-scores < -3 SD) and 73.1% (n = 68) as moderately thin (Z-scores between -3 SD and -2 SD), according to the WHO Growth Reference 2007 [48–50] (Table 2).

**Table 2:** Nutritional status of adolescents living with HIV on ART follow-up in Ethiopia based on anthropometric measurements (n = 384).

Measurement Variable	Description	Frequency n (%)
BMI-for-Age Z-score (BAZ)	Thin	93 (24.2%)
	Normal/Not Thin	291 (75.8%)
Height-for-Age Z-score (HAZ)	Stunted	83 (21.6%)
	Normal/Not stunted	301 (78.4%)
Mid-Upper Arm Circumference (MUAC)	Acutely Malnourished	134 (34.9%)
	Normal/ Not Acutely Malnourished	250 (65.1%)

**Note:**

• **Thinness categories:** BAZ < -2 SD: Thin; BAZ ≥ -2 SD: Not Thin.

• **Stunting categories:** HAZ < -2 SD: Stunted; HAZ ≥ -2 SD: Not Stunted.

• **Acute Malnutrition (MUAC) categories:** MUAC < 18.5 cm for 10–14 years or < 21 cm for 15–19 years: Acutely Malnourished; MUAC ≥ 18.5 cm for 10–14 years or ≥ 21 cm for 15–19 years: Normal/ Not Acutely Malnourished.

For stunting, 21.7% (n = 83) of participants exhibited stunted growth (HFA Z-scores < -2 SD), with 28.9% (n = 24) classified as severely stunted (Z-scores < -3 SD) [51] (Table 2).

For acute malnutrition, 34.9% (n = 134) of participants were identified as malnourished based on MUAC measurements [52,53]. Among these, 80.6% (n = 108) had moderate acute malnutrition, and 19.4% (n = 26) were classified as having severe acute malnutrition (Table 2).

#### 3.1.3. Clinical characteristics.

**HIV-related information:** The median age at HIV diagnosis and ART initiation was 3.95 years (mean 4.66 ± 3.7 years). More than half of the participants (50.5%, n = 194) initiated ART before the age of 4 years. Most participants (63%, n = 242) had started ART 15 years ago or more. A majority (79.9%, n = 307) had known their HIV status for four or more years. Nearly half (47.4%, n = 182) had a history of missed ART clinic appointments (S1 Table).

A significant majority (81.8%, n = 314) of the study participants had at least one family member who was HIV-positive in their households. Among these, 52.9%(n = 166) reported that only one parent was HIV-positive, and 8.3%(n = 26) had a single parent affected, 24.6% (n = 76) had both parents living with HIV, and 13.9%(n = 43) reported the entire nuclear family (parents and sibling/s) were HIV-positive.

Almost all participants (98.7%, n = 379) had undergone viral load assessment within the previous three months. Viral load data were unavailable for 1.3% of participants because laboratory results were not documented in the clinical records at the time of data extraction. Among those with available results, 97.7% had a viral load test result of less than 150 copies/ ml, and only 1% had 150 copies/ml or more. At ART enrolment, half of the participants had a CD_4_ level of 500–1500 cells/mm^3^, and 36.7% (n = 141) had a CD_4_ level below 500 cells/mm^3^ at the time of data collection. Before starting ART, 64.3% (n = 247) were in clinical Stage I and 29.4% (n = 113) were in Stage III. After ART initiation, most participants (96.6%) were in clinical Stage I, indicating considerable improvement. Most study participants (79.4%, n = 305) were taking first-line anti-retroviral therapy (ART) (S2 Table).

**Fig 1:**
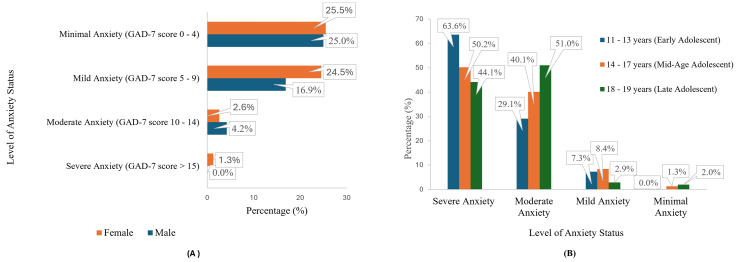
Prevalence of anxiety assessed using the Generalized Anxiety Disorder 7-item scale (GAD-7) by gender (A) and age category (B) among ALHIV on ART follow-up in Ethiopia 2024 (n = 384).

**Mental health:** *Anxiety.* Nearly half of the participants (49.5%, n = 190) reported symptoms of anxiety according to the GAD-7 scale. Of these, 8.1% (n = 41) had moderate to severe anxiety, while 41.4% (n = 159) experienced mild anxiety. Female participants reported higher levels of anxiety (28.4%, n = 109) than males (21.1%, n = 81), though the difference was not statistically significant (Fig 1A). Anxiety prevalence was higher among older adolescents, with 36.4% of early adolescents (10–13 years), 49.8% of mid-adolescents (14–17 years), and 55.9% of late-age adolescents (18–19 years) experiencing mild to severe anxiety (Fig 1B).

**Fig 2:**
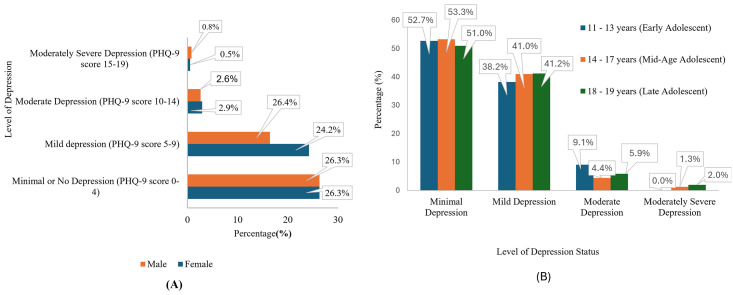
Distribution of depression assessed using a modified PHQ-9 by gender (A) and age category (B) among ALHIV on ART follow-up in Ethiopia 2024 (n = 384).

*Depression.* Symptoms of depression, measured by the PHQ-9 scale, were reported by 27.6% (n = 106) of participants, with 6.8% (n = 26) experiencing moderate to severe depression. A higher proportion of female (27.6%, n = 106) reported depressive symptoms compared to males (19.8%, n = 76) (Fig 2A). Depression rates were similar across age groups, with 47.3% of early- adolescents, 46.7% of mid-adolescents, and 49.0% of late-adolescents reporting mild to moderate depressive symptoms (Fig 2B).

**Other health problems:** Most participants (81.5%; n = 313) had experienced opportunistic infections at some point in their lives. Among these, 57.5% (n = 221) reported chronic respiratory illnesses such as colds, pneumonia, and tuberculosis; notably, 33.1% (n = 127) had a history of tuberculosis and had received an anti-tuberculosis treatment. Other common health problems included gastrointestinal issues (56.5%, n = 217; e.g., salmonellosis, gastritis/peptic ulcer disease), dermatologic conditions (46.4%, n = 178), and central nervous system problems (33.3%, n = 128; e.g., headaches, meningitis, epilepsy, Guillain-Barré Syndrome). Half of the participants reported experiencing some form of illness in the previous three months. Among these, 67.2% had respiratory system problems and 19.3% (n = 74) had gastrointestinal system issues (S1 Table).

#### 3.1.4. Lifestyle-related characteristics - Alcohol use.

Overall, 29.9%(n = 115) of participants reported ever using alcohol, and 9.9% (n = 38) reported current alcohol consumption. Among current users, 34.2% (n = 13) exhibited risky, high-risk, or addictive drinking patterns (Table 3). Risky alcohol use was more common among males (26.4%, n = 10) than females (7.9%, n = 3). Risky or addictive drinking was more prevalent among late adolescents (18–19 years:23.7%, n = 9), with 7.9% (n = 3) classified as high-risk. Among mid-adolescents (14–17 years), 10.5% (n = 4) displayed risky drinking behaviours.

**Table 3:** Alcohol consumption risk category of ALHIV on ART follow-up in Ethiopia using the *AUDIT screening test, 2024 (n = 38).

Variables	Frequency n (%)
Low risk (AUDIT Score 1 – 7)	25 (65.8%)
Risky (AUDIT Score 8 – 15)	10 (26.3%)
High Risk (AUDIT Score 16–19)	1 (2.6%)
Addiction Likely (AUDIT Score 20–40)	2 (5.3%)

*AUDIT - Alcohol Use Disorders Identification Test.

Qualitative responses indicated that alcohol use was often linked to peer pressure and coping with stress.

*“Sometimes my friends encourage me to drink, and it helps me to forget my worries for a while” (Male, 17).*

Statistical analysis did not find a significant association between alcohol use and missed ART appointments or self-reported adherence in this sample.

#### 3.1.5. Environmental issues and dietary considerations.

Food insecurity was a major concern among participants. Approximately one-third (34.4%, n = 132) reported insufficient food availability at home, while over half (53.1%, n = 204) reported limited variety of foods due to financial constraints. More than half (54.2%, n = 208) - reported that they could not consume their preferred foods, and 40.1% (n = 154) often ate foods they disliked or consumed smaller portions because of scarcity. Nearly half (44.8%, n = 172) ate fewer meals per day than desired, and 25.8% (n = 99) reported periods with no food available at home. More than half (55.3%, n = 212) went to bed hungry at least once per week, and 15.4% (n = 59) experienced a full day and night without food. Overall, 83.6% (n = 321) reported skipping meals, and 53.1% (n = 204) lived in food-insecure households (S3 Table).

Household food preservation practices were common: 91.9%(n = 353) used canning and 79.9% (n = 307) used drying. About half also practiced refrigeration, freezing, and salting to prolong food shelf life.

Access to safe water and sanitation was relatively high but unevenly distributed. Most participants (77.3%; n = 297) had access to clean water available at home. Among those without direct access (22.7%, 87), nearly half (48.3%, n = 42) reported treating their water using chlorine-based solutions or water filters. The majority (82%, n = 315) used pit latrines, while 17.2% (n = 66) had flush toilets; only one participant (0.3%) reported open-field defecation (S4 Table).

Feeding-related health issues were frequent, with 28.6% (n = 110) reporting vomiting, diarrhea, or loss of appetite. Nutritional supplementation was provided to 62% (n = 238), mainly through Ready-to-Use Therapeutic Food (RUTF) distributed at ART clinic. More than half of those supplemented received one 92g sachet daily for up to two months (S1 Table).

Qualitative responses highlighted that limited household income, stigma, and illness often constrained food choices and intake. Adolescents noted:

*“Sometimes we only eat what is available, even if it’s not what we want. When there’s no money, we just skip meals.” (Female, 16)*

*“When my mother is sick and cannot work, we depend on neighbours for food. It is hard to eat well every day.” (Male 14)*

Other participants described social challenges of food insecurity at school:

*“I feel shy to eat at school because others bring good food and I don’t. Sometimes I just drink water.” (Female, 15)*

These quotes illustrate the emotional and social dimensions of food insecurity experienced by adolescents living with HIV, complementing the quantitative findings and emphasizing the contextual barriers to adequate nutrition.

### 3.2. Factors associated with malnutrition

#### 3.2.1. Factors associated with thinness.

A regression analysis was conducted to explore factors associated with malnutrition defined as thinness (i.e., BMI-for-age Z-score values below -2 SD). Adjusted odds ratios (AORs) with 95% confidence intervals (Cis) were used to estimate the strength of associations. Potential variables for inclusion in the model were initially identified using correlation analysis (S5 Table). Ten variables met the inclusion criterion of p-value < 0.25 in univariate analysis. These included sociodemographic factors (age, sex/gender, household monthly income), clinical and HIV-related characteristics (history of nutritional assessment during ART initiation, duration of HIV awareness, history of chronic infections such as tuberculosis, anxiety symptoms measured by GAD-7, depression symptoms measured by PHQ-9, and environmental and dietary factors (food insecurity concern, history of nutritional supplement use).

**Sociodemographic variables:** Among the sociodemographic variables analysed, only sex was significantly associated with thinness. Female adolescents living with HIV (ALHIV) had 73% lower odds of being thin compared to male counterparts (AOR = 0.27; 95% CI: 0.16, 0.45). Age and household monthly income were not significantly associated with thinness (Table 4).

**Table 4:** Factors significantly associated with thinness/malnutrition (BAZ) among ALHIV on ART in Ethiopia, 2024 (n = 384).

Variables	Category	Nutritional status		COR (95% CI)	AOR (95% CI)
		Thin/ malnourished (BAZ < -2SD)	Normal (BAZ ≥ -2 SD)		
		n (%)	n (%)		
**(a) Sociodemographic characteristics**					
Age	Early adolescent age	9 (2.3)	46 (15.8)	0.57 (0.27, 1.22)	
	Mid and late adolescent age	84 (21.3)	245 (63.8)	1	
Sex	Male	109 (28.4)	98 (25.5)	1	1
	Female	131 (34.1)	46 (12.0)	0.27 (0.16, 0.44) ***	0.27(0.16, 0.45) ***
Family monthly income	< 3000 EBR	128 (33.3)	51 (13.3)	1	
	≥ 3000 EBR	56 (14.6)	55 (14.3)	0.20 (0.74, 1.18)	
**(b) Clinical Variables**					
Duration aware of HIV status	≤ 3 years ago,	24 (6.3)	53 (13.8)	1.56 (0.9, 2.71)	
	≥ 4 years ago,	69 (18.0)	238 (62.0)	1	
Ever had long-lasting infections such as tuberculosis	Yes	41 (10.7)	86 (22.4)	1	1
	No	52 (13.5)	205 (53.4)	0.53 (0.33, 0.86) **	0.48 (0.29, 0.80) **
GAD scores (anxiety)	Yes	56 (14.6)	134 (34.9)	1	1
	No	37 (9.6)	157 (40.9)	0.56 (0.35, 0.91) **	0.53 (0.32, 0.88) **
PHQ-9 scores (depression)	Moderate to moderately severe depression scores	111 (2.9)	15 (3.9)	1	1
	Minimal to mild depression scores	82 (21.4)	276 (71.9)	0.41 (0.18, 0.92) *	0.40 (0.17, 0.91) *
**(c) Environmental and Dietary related factors**					
Ever had food insecurity concerns in the household	No	52 (13.5)	127 (33.1)	1	1
	Yes	41 (10.7)	164 (42.7)	1.64 (1.02, 2.62) *	1.63 (1.02, 2.62) *
Ever had a nutritional assessment during ART initiation	No	24 (6.3%)	122 (31.8%)	1	
	Yes	69 (18.0%)	169 (44.0%)	0.63 (0.36, 1.10)	
Ever had nutrition supplementation intake	No	83 (21.6)	63 (16.4)	1	1
	Yes	157 (40.9)	81 (21.1)	0.48 (0.29, 0.81) *	0.55 (0.32, 0.95) *

Significant at *P-value <0.05, **P-value <0.01 and ***P-value<0.001; AOR: Adjusted Odds Ratio, COR: Crude Odds Ratio, GAD: Generalized Anxiety Disorder; PHQ-9: Patient Health Questionnaire-9.

**Clinical variables:** ALHIV who had never experienced chronic infections, such as tuberculosis, had 52% lower odds of being thin compared to those with a history of such infections (AOR = 0.48; 95% CI: 0.29, 0.80). This observed association highlights the potential link between chronic infection and malnutrition, which may be bidirectional (Table 4).

Adolescents without symptoms of anxiety (GAD-7 < 5) had 47% lower odds of being thin compared to those with higher anxiety scores (AOR = 0.53; 95% CI: 0.32, 0.88). Similarly, participants with minimal to mild depression (PHQ-9 scores < 9) had 60% lower odds of being thin than those with moderate to severe depression scores (AOR = 0.40; 95% CI: 0.17, 0.91). History of nutritional assessment during ART initiation and duration of HIV awareness were not significantly associated with thinness (Table 4).

**Lifestyle factor:** Alcohol consumption was not significantly associated with thinness in this cohort.

**Environmental and dietary factors:** Food insecurity was associated with higher odds of thinness. ALHIV experiencing food insecurity had 1.63 times higher odds of being thin compared to those without food insecurity (AOR = 1.63; 95% CI: 1.02, 2.62). Conversely, adolescents who received nutritional supplementation had 45% lower odds of thinness compared to those who did not receive supplements (AOR = 0.55; 95% CI: 0.32, 0.95). No other environmental and dietary factors were significantly associated with thinness (Table 4).

#### 3.2.2. Factors associated with acute malnutrition.

A regression analysis was conducted to identify factors associated with acute malnutrition, defined as mid-upper arm circumference (MUAC) < 18.5 cm for adolescents aged 10–14 years or < 21 cm for those aged 15–19 years (S1 Text). Correlation analysis was conducted to determine the potential variables for entry in the model (S6 Table). Thirteen variables with p-value < 0.25 in the univariate analysis were included in the multivariate model. These comprised sociodemographic factors (age, sex, family size), clinical characteristics (anxiety status, haemoglobin, alanine aminotransferase (SGPT) level, duration of HIV awareness, history of long-term illness), and environmental and dietary factors (household food insecurity, number of meals consumed in 24 hours, and history of nutrition supplementation).

**Sociodemographic variables:** Early and mid-adolescent participants had higher odds of experiencing acute malnutrition than late-adolescents. The odds of malnutrition were nearly two times higher among adolescents aged 10–17 years (AOR = 1.94; 95% CI: 1.03, 3.64). Female adolescents had 42% lower odds of being malnourished than males (AOR = 0.58; 95% CI: 0.38, 0.88). In addition, adolescents living in smaller households (≤ 3 members) had 56% lower odds of experiencing malnutrition compared to those in larger households (≥4 members) (AOR = 0.44; 95% CI: 0.24, 0.81) (Table 5).

**Table 5:** Factors associated with acute malnutrition using MUAC-for-age indices among ALHIV on ART in Ethiopia, 2024 (n = 384).

Variables	Category	Nutritional Status using MUAC-for-Age indices		COR (95% CI)	AOR (95% CI)
		Acutely Malnourished	Not acutely Malnourished		
		n (%)	n (%)		
(a) Sociodemographic Characteristics					
Age	Early and mid-adolescent age (10–17 years)	106 (27.6)	176 (45.8)	1.59 (0.97, 2.62) *	1.94 (1.03, 3.64) *
	Late adolescent age (18–19 years)	28 (7.3)	74 (19.3)	1	1
Sex	Female	61 (15.9)	146 (38.0)	0.59 (0.39, 0.91) *	0.58 (0.38, 0.88) *
	Male	73 (19.0)	104 (27.1)	1	1
Household monthly income	< 3000 EBR	82 (21.4)	134 (34.9)	1.36 (0.89, 2.09)	
	≥ 3000 EBR	52 (13.5)	116 (30.2)	1	
Family size	Less than or equal to three	35 (9.1)	95 (24.7)	0.58 (0.36, 0.92) *	0.44 (0.24, 0.81) *
	Four and above	99 (25.8)	155 (40.4)	1	1
(b) Clinical Variables					
Duration aware of HIV status	≤ 3 years ago	42 (10.9)	35 (9.1)	2.80 (1.68, 4.67) ***	2.96 (1.62. 5.40) ***
	≥ 4 years ago	92 (24.0)	215 (56.0)	1	1
PHQ-9 scores (depression)	Minimal/no depression scores	64 (16.7)	138 (35.9)	0.74 (0.49, 1.13)	
	Mild to severe depression scores	70 (18.2)	112 (29.2)	1	
GAD scores (anxiety)	No	58 (15.1)	136 (35.4)	0.64 (0.42, 0.98) *	0.50 (0.28, 0.89) *
	Yes	76 (19.8)	114 (29.7)	1	1
Ever had longstanding disease (e.g., tuberculosis)	No	57 (14.8)	70 (18.2)	0.53 (0.34, 0.82) **	0.52 (0.33, 0.81) **
	Yes	77 (20.1)	180 (46.9)	1	1
Haemoglobin status	<11.5 gm/dl	26 (7.2)	29 (8.1)	3.59 (1.06, 12.12) *	7.29 (1.31, 40.5) *
	11.5–15.0 gm/dl	98 (27.3)	186 (51.8)	1.70 (0.95, 3.05)	1.92 (0.83, 4.42)
	>15.0 gm/dl	4 (1.1)	16 (4.5)	1	1
Alanine transaminase (SGPT)	0–50 U/L	86 (36.8)	135 (57.7)	0.40 (0.13, 1.26) *	0.29 (0.09, 0.99) *
	>50 U/L	8 (3.4)	5 (2.1)	1	1
(c) Environmental and Dietary related factors					
Ever had food insecurity concerns in households	Yes	64 (16.7)	141 (28.4)	1.46 (0.96, 2.22) *	1.85 (1.09, 2.14) *
	No	70 (18.2)	109 (28.4)	1	1
Number of meals eaten in 24 hours (day and night)	≤ 2 meals	30 (7.8)	30 (7.8)	2.11 (1.21, 3.69) *	1.86 (1.08, 3.19) *
	3–5 meals	104 (27.1)	220 (57.3)	1	1
Ever had nutrition supplementation intake	No	39 (10.2)	107 (27.9)	0.55 (0.35, 0.86) **	0.45 (0.36, 0.79) **
	Yes	95 (24.7)	143 (37.2)	1	1

Significant at *P-value <0.05, **P-value <0.01 and ***P-value<0.001; AOR: Adjusted Odds Ratio, COR: Crude Odds Ratio, GAD: Generalized Anxiety Disorder; PHQ-9: Patient Health Questionnaire-9.

**Clinical variables:** Adolescents without anxiety symptoms (GAD-7 score < 5) had lower odds of acute malnutrition than those with higher anxiety scores (GAD-score ≥5) (AOR = 0.50; 95% CI: 0.28, 0.89). Adolescents who had been aware of their HIV status for three years or less had nearly threefold higher odds of malnutrition (AOR = 2.96; 95% CI: 1.62, 5.40).

Low haemoglobin levels (<11.5 g/dl) were associated with higher odds of malnutrition compared with haemoglobin levels > 15g/dl (AOR = 7.29; 95% CI: 1.31, 40.5). Conversely, adolescents with SGPT levels within normal range (0–50 U/L) had lower odds of being malnourished (AOR = 0.29; 95% CI: 0.09, 0.99).

Participants without a history of prolonged illness, such as tuberculosis, had lower odds of experiencing malnutrition (AOR = 0.52; 95% CI: 0.33, 0.81). This association highlights a potential bidirectional link between chronic illness and malnutrition, consistent with previous evidence in similar populations (Table 5).

**Environmental and dietary-related factors:** Adolescents experiencing food insecurity had higher odds of acute malnutrition than those from food-secure households (AOR = 1.85; 95% CI: 1.09, 2.14). Similarly, those who reported consuming two or fewer meals per day had 1.86 times higher odds of malnutrition compared to those who consumed three or more meals daily (AOR = 1.86; 95% CI: 1.08, 3.19). Conversely, adolescents who received nutritional supplementation had lower odds of being malnourished (AOR = 0.45; 95% CI: 0.36, 0.79) (Table 5).

#### 3.2.3. Factors associated with stunting.

We assessed the association of stunting with sociodemographic, clinical, lifestyle, and environmental and dietary factors. None of these factors were statistically significantly associated with stunting among adolescents living with HIV (all p-value > 0.05).

In the qualitative component, participants described experiences of food insecurity, limited household resources, and social and emotional barriers affecting their nutritional intake. As described in Section 3.1.5, adolescents reported skipping meals when food was scarce and feeling uncomfortable eating at school because of perceived social stigma.

## 4. Discussion

Adolescence represents a crucial stage for physical and psychological development, during which nutritional adequacy is essential for healthy growth and optimal ART outcomes [54]. This study revealed substantial levels of malnutrition among adolescents living with HIV in Ethiopia, including thinness (24.2%), stunting (21.7%), and acute malnutrition (34.9%). Notably, nearly one-fifth of the malnourished adolescents were classified as having severe acute malnutrition based on MUAC, underscoring the presence of clinically severe nutritional deficits within routine HIV care settings. These findings highlight the continuing burden of undernutrition in the vulnerable population despite national and global efforts to strengthen HIV care and adolescent nutrition programs.

Adolescence represents a crucial stage for physical and psychological development, during which nutritional adequacy is essential for healthy growth and optimal ART outcomes [54]. This study revealed substantial levels of malnutrition among adolescents living with HIV in Ethiopia, including thinness (24.2%), stunting (21.7%), and acute malnutrition (34.9%). Notably, nearly one-fifth of the malnourished adolescents were classified as having severe acute malnutrition based on MUAC, underscoring the presence of clinically severe nutritional deficits within routine HIV care settings. These findings highlight the continuing burden of undernutrition in the vulnerable population despite national and global efforts to strengthen HIV care and adolescent nutrition programs. Several factors were significantly associated with malnutrition among adolescents living with HIV in this study, including male sex, younger age, larger household size, food insecurity, chronic infections such as tuberculosis, symptoms of anxiety and depression, low haemoglobin levels, and inadequate meal frequency. Conversely, nutritional supplementation and absence of chronic illness or anxiety symptoms were associated with lower odds of malnutrition. These findings highlight the multifactorial nature of malnutrition among adolescents receiving ART and underscore the need for integrated clinical, nutritional, and psychosocial interventions.

The prevalence of thinness (24.2%) among adolescents in this study was comparable to reports from Uganda and southern Ethiopia among similar populations of ALHIV [14,55]. However, it was higher than findings from Nigeria and more recent Ugandan studies conducted in general adolescent populations, which may have lower underlying risk of malnutrition [56,57]. These differences likely reflect variations in study populations, socio-economic conditions, healthcare access, and HIV-related nutritional vulnerabilities. Similarly, the prevalence of stunting (21.7%) is consistent with recent Ugandan studies(25%) [57], but lower than reported in southern Ethiopia (33.1%), and earlier Ugandan research (36%) [14,55,58]. These variations may reflect contextual differences in socio-economic conditions, healthcare access, and dietary patterns. Although the relatively lower rate of stunting suggests some improvements in nutritional support and healthcare services, the persistence of both thinness and stunting highlights an ongoing public health concern. Addressing the underlying socio-economic and environmental factors remains critical for improving nutritional outcomes and tackling malnutrition among adolescents living with HIV.

These findings highlight the multifaceted nature of malnutrition among ALHIV. Age, gender, and family size showed significant association with nutritional status. Younger adolescents (aged 10–17 years) were more likely to experience acute malnutrition, likely reflecting the heightened nutritional demands of puberty combined with HIV-related illness and potential ART side effects [15,50,59]. The rapid physical and cognitive growth characteristics in this stage may further increase vulnerability when dietary intake is inadequate. These findings emphasize the importance of age-appropriate nutritional interventions to prevent growth faltering and improve overall health among adolescents. Given that most participants were students, school-based programs may serve as an effective platform to deliver nutritional counselling, dietary supplementation, and health education [60].

Gender-based differences were also observed, with male adolescents showing a higher likelihood of malnutrition than females. In this study, male participants had significantly higher odds of being thin and acutely malnourished compared with female participants, while females had 42–73% lower odds of malnutrition across the models, highlighting male sex as a key determinant of nutritional vulnerability among ALHIV. This pattern is consistent with studies from sub-Saharan Africa reporting higher rates of undernutrition among male adolescents living with HIV, potentially reflecting increased energy requirements during puberty, reduced dietary intake due to appetite loss or treatment side effects, and lower engagement with health and nutrition services among males. In contrast, female adolescents may benefit from greater health-seeking behaviour or household prioritization of food needs. These findings underscore the importance of gender-responsive approaches within adolescent HIV care that address the nutritional vulnerabilities of male adolescents while sustaining support for females.

Household size also showed an association with nutritional status, where adolescents from smaller families had better nutritional outcomes. This may be due to greater resource availability per household member, facilitating access to adequate and diverse foods. Although household income was not directly linked to malnutrition in this study, the qualitative findings suggested that limited resources and competing priorities within larger households constrained food access and diversity. These findings reinforce the importance of addressing broader social determinants such as household structure and economic capacity when designing nutritional interventions for ALHIV.

Clinical and biological factors were associated with malnutrition. Adolescents with a history of chronic infections, such as tuberculosis, were linked to higher odds of malnutrition. This relationship is likely bidirectional: infections can exacerbate nutritional deficits, while malnutrition may increase susceptibility to infection [61,62]. Similarly, low haemoglobin levels were associated with malnutrition, consistent with evidence that anaemia and undernutrition frequently co-occur in the HIV-infected populations [63]. However, this cross-sectional design precludes establishing directionality; malnutrition may contribute to anemia through micronutrient deficiency, while anemia may also arise from infection or ART side effects. Normal liver enzyme (SGPT) levels appeared protective, potentially reflecting better overall metabolic health, whereas elevated levels may indicate hepatic dysfunction that may compound nutritional risk [64]. These findings highlight the need for comprehensive healthcare management for ALHIV, including regular monitoring and targeted intervention for infections, anaemia, and liver dysfunction, to improve nutritional outcomes and overall health.

Food insecurity and dietary practices were significantly linked with nutritional outcomes. Adolescents with limited meal frequency and low dietary diversity were more likely to experience thinness and acute malnutrition. Those experiencing food insecurity were more likely to be thin, echoing previous findings linking food insecurity to poor nutritional outcomes among HIV-positive individuals [14,65]. Conversely, nutritional supplementation and consumption of three or more meals per day were associated with lower risk of malnutrition. These results underscore the importance of targeted nutritional programs integrated as part of comprehensive HIV care. Dietary patterns also played a vital role, as consuming three or more meals per day reduced malnutrition risk, emphasising the importance of promoting consistent healthy eating practices and improving access to nutritious foods [66]. Participants’ qualitative responses illustrated how limited household resources and social constraints shaped their access to sufficient and preferred foods, further highlighting the psychosocial dimensions of food insecurity and the need to integrate household- and community-based interventions.

Environmental conditions, such as limited access to clean water and sanitation, were associated with increased susceptibility to infections, further compromising nutritional status [67–70] This underscores the need to improve water and sanitation infrastructure to promote better health outcomes among ALHIV. Overall, these findings suggest that addressing malnutrition in this population requires comprehensive strategies targeting both immediate nutritional needs and underlying socio-economic and health factors.

Interestingly, no factors were found to be significantly associated with stunting (HFA indices), highlighting an area for future research. This finding suggests that linear growth deficits in ALHIV may result from cumulative or long-term influences not captured in this cross-sectional study, such as early childhood malnutrition, genetic predispositions, or prolonged ART exposure. Unlike thinness and acute malnutrition, which reflected current dietary and clinical conditions, stunting likely represents chronic nutritional challenges. Future longitudinal studies should examine early-life nutrition, long-term ART effects, and growth trajectories to better understand the factors associated with stunting in this population.

Although no statistically significant associations were observed in the quantitative analysis for stunting, the qualitative findings suggest that broader social and environmental constraints, including food insecurity and stigma-related barriers to eating, may contribute to chronic undernutrition and impaired linear growth. This discrepancy highlights the value of mixed-methods approaches in capturing determinants of stunting that may not be detected through cross-sectional quantitative models alone.

In summary, the findings suggest that food insecurity, poor dietary diversity, inadequate healthcare, clinical factors, socio-economic conditions and environmental constraints significantly affect the nutritional outcomes of ALHIV, contributing to the high prevalence of malnutrition observed in this population. These underlying issues may further exacerbate the nutritional deficiencies observed among ALHIV, who face compounded health challenges due to their HIV status. Addressing these disparities requires a holistic approach that integrates improved nutrition, enhanced healthcare access, and targeted support for ALHIV. Given that nearly all participants in this study were students, school-based programs that integrate HIV management and nutritional care may offer an effective strategy to improve adolescent health outcomes, as demonstrated in other adolescent health interventions [71–73].

### 4.1. Limitations

This study encountered several limitations that may influence the interpretation of its findings. First, the reliance on self-reported data from adolescents living with HIV (ALHIV) may have introduced recall, response, or social desirability bias, potentially affecting the accuracy of participants’ reports of dietary practices and psychosocial experiences. Although triangulation and standardized data collection protocols were employed to minimize these effects, their influence on data accuracy cannot be ruled out. Future research could enhance reliability by incorporating objective measures, such as biomarker assessments or digital dietary monitoring, and by applying cross-validation techniques.

Second, excluding caregiver perspectives may have limited understanding of the broader household and social dynamics influencing adolescent nutrition. Caregivers play a pivotal role in food provision, treatment adherence, and emotional support, and their insights into food security and healthcare access could provide a more comprehensive view of factors shaping nutritional outcomes among ALHIV. Future studies should therefore include caregiver input to strengthen contextual understanding.

Third, the cross-sectional design precludes establishing causal relationships between nutritional practices, health outcomes, and psychosocial factors. The observed associations should be interpreted as correlational rather than causal. Longitudinal or interventional studies are needed to better explore the temporal and causal pathways linking these factors.

Lastly, the study was conducted in two regions of Ethiopia during a period marked by socio-political and economic challenges, as well as disruptions caused by the COVID-19 pandemic. These factors may have influenced both health service delivery and household food availability. Future research with larger and more geographically diverse samples, using longitudinal designs and improved data collection methods, could provide more robust evidence on the long-term impacts of nutritional and healthcare interventions for ALHIV.

## 5. Conclusion and recommendations

This study revealed a substantial burden of malnutrition – manifested as thinness, stunting, and acute malnutrition – among adolescents living with HIV (ALHIV). These findings highlight the ongoing nutritional and health vulnerabilities within this population, despite advances in ART coverage and adolescent care programs. Malnutrition among ALHIV arises from an interplay of socio-demographic, clinical, environmental, and psychosocial factors, emphasizing the need for integrated nutritional support and healthcare interventions that address these interrelated factors.

The findings reinforce the importance of age- and gender-specific interventions, particularly for younger adolescents and male ALHIV, who are at higher risk of malnutrition. Tailored programs that account for developmental and gender differences may improve long-term health outcomes. Addressing food insecurity, promoting dietary diversity, and encouraging regular meal consumption are also critical strategies to improve nutritional status.

The observed association between chronic infections, anaemia, and liver dysfunction with malnutrition underscore the need for comprehensive healthcare management. Routine screening, early detection, and timely treatment of these conditions should be integrated into HIV care. Enhancing access to safe water and improving sanitation can further reduce the environmental risks that contribute to malnutrition among ALHIV.

To optimize health and nutritional outcomes, healthcare programs should integrate nutritional support, management of chronic infections, and mental health services within adolescent-friendly and gender-responsive models of care. Healthcare workers should be trained to deliver holistic, developmentally appropriate care that addresses the unique needs of adolescents. Future research should employ longitudinal designs to elucidate causal pathways and evaluate long-term effectiveness of integrated interventions.

Finally, integrating HIV management and nutritional support within school health programs could expand access to services for adolescents. Concurrently, policy initiatives aimed at reducing socio-economic disparities remain essential to fully address the nutritional and health needs of ALHIV.

## Supporting information

S1 Text Operational definitions. (DOCX)

S2 Text Probability proportional to size (PPS) allocation of adolescents living with HIV across participating hospitals. (DOCX)

S1 Table Clinical factors: HIV-related characteristics of adolescents living with HIV on ART follow-up in Ethiopia, 2024 (n = 384). (DOCX)

S2 Table Medical record profile of adolescents living with HIV on ART follow-up in Ethiopia, 2024 (n = 384). (DOCX)

S3 Table Environmental and dietary factors: household food availability, supplementation, and dietary intakes of adolescents living with HIV on ART follow-up in Ethiopia, 2024 (n = 384). (DOCX)

S4 Table Environmental factors: household water and toilet facility-related characteristics of adolescents living with HIV on ART follow-up in Ethiopia, 2024 (n = 384). (DOCX)

S5 Table Correlation analysis to determine variables for inclusion in modelling for thinness. (DOCX)

S6 Table Correlation analysis to determine variables for inclusion in modelling for acute malnutrition. (DOCX)
