# Supplementary material for: Clinical, lifestyle, environmental and dietary determinants of malnutrition in adolescents on antiretroviral therapy in Ethiopia
Source: PLOS Glob Public Health. 2026 Jun 26;6(6):e0005003. doi: 10.1371/journal.pgph.0005003 (PMC13309033; doi:10.1371/journal.pgph.0005003)
Supplement: S1 Text — (DOCX) [file pgph.0005003.s001.docx]

**Supporting Information**

| **S1 Text. Operational definitions** |
| --- |
| The comprehensive evaluation of nutritional status involved collecting medical history, dietary history, performing a physical examination, conducting anthropometry, performing laboratory tests, and collecting lifestyle data. It included weight, height, BMI, MUAC, skinfold thickness, body fat percentage, biochemical analyses (e.g., haemoglobin, vitamins), clinical signs of malnutrition, and dietary intake  **Body Mass Index (BMI)** was calculated as weight in kilograms divided by height in meters squared (kg/m²). Collapsed into categories, these included underweight (BMI < 18.5 kg/m²), normal weight (18.5–24.9 kg/m²), overweight (25–29.9 kg/m²), and obesity (≥30 kg/m²) based on WHO (2006) standards.  **Malnutrition** encompassed undernutrition, micronutrient deficiencies, and overweight or obesity.  For ALHIV aged 10–14 years, severe malnutrition was indicated by MUAC <16 cm, moderate malnutrition by MUAC 16–18.49 cm, and normal status by MUAC ≥18.5 cm.  For ALHIV aged 15–19 years, severe malnutrition was defined as MUAC <18.5 cm, moderate as MUAC 18.5–21 cm, and normal as MUAC ≥21 cm (Kristen & Lesle, 2018)  **Stunting** was defined by a height-for-age Z-score below -2 SD, with severe stunting indicated by a Z-score below -3 SD (De Onis et al, 2007; FANTA-III, 2013; WHO, 2007). **Thinness** referred to BMI-for-age Z-score values below -2 SD, with severe thinness defined by a Z-score below -3 SD (FANTA-III, 2013; WHO, 2007). |
