# Supplementary material for: Clinical, lifestyle, environmental and dietary determinants of malnutrition in adolescents on antiretroviral therapy in Ethiopia
Source: PLOS Glob Public Health. 2026 Jun 26;6(6):e0005003. doi: 10.1371/journal.pgph.0005003 (PMC13309033; doi:10.1371/journal.pgph.0005003)
Supplement: S2 Text — (DOCX) [file pgph.0005003.s002.docx]

**Supporting Information**

**S2 Text. Probability proportional to size (PPS) allocation of adolescents living with HIV across participating hospitals.**

| \| **No** \| **Hospital** \| **ALHIV on ART enrolment (Ni)** \| **PPS Allocation** \| \| --- \| --- \| --- \| --- \| \| 1 \| Shashemene Comprehensive Specialized Hospital \| 70 \| 14 \| \| 2 \| Batu Hospital \| 11 \| 2 \| \| 3 \| Assela Hospital \| 126 \| 24 \| \| 4 \| Bishoftu Hospital \| 206 \| 40 \| \| 5 \| Adama Hospital \| 259 \| 50 \| \| 6 \| Yekatit 12 Hospital \| 274 \| 53 \| \| 7 \| Zewditu Hospital \| 284 \| 55 \| \| 8 \| St Paulo’s Specialized Hospital \| 375 \| 72 \| \| 9 \| ALERT Specialized General Hospital \| 372 \| 72 \| \| 10 \| Ras Desta Hospital \| 12 \| 2 \| \|  \| **Total** \| **1989** \| **384** \| |
| --- | --- | --- | --- | --- | --- | --- | --- | --- | --- | --- | --- | --- | --- | --- | --- | --- | --- | --- | --- | --- | --- | --- | --- | --- | --- | --- | --- | --- | --- | --- | --- | --- | --- | --- | --- | --- | --- | --- | --- | --- | --- | --- | --- | --- | --- | --- | --- | --- |
