# Supplementary material for: Clinical, lifestyle, environmental and dietary determinants of malnutrition in adolescents on antiretroviral therapy in Ethiopia
Source: PLOS Glob Public Health. 2026 Jun 26;6(6):e0005003. doi: 10.1371/journal.pgph.0005003 (PMC13309033; doi:10.1371/journal.pgph.0005003)
Supplement: S1 Table — (DOCX) [file pgph.0005003.s003.docx]

**Supporting Information**

**S1 Table. Clinical factors: HIV-related characteristics of adolescents living with HIV on ART follow-up in Ethiopia, 2024 (n=384)**

| Variables | Description | Frequency N (%) |
| --- | --- | --- |
| Time when became aware of HIV status | ≤ 3 years ago | 77 (20.1) |
|  | ≥ 4 years ago | 307 (79.9) |
| Has HIV-positive family member | Yes | 309 (80.5) |
|  | No | 59 (15.4) |
|  | Unknown | 16 (4.2) |
| Family member(s) who are HIV positive [*multiple response possible] (n=314) | Only Father | 26 (8.3) |
|  | Only Mother | 166 (52.9) |
|  | Both Mother and Father | 76 (24.2) |
|  | Nuclear Family [Mother, Father, Brother and/or Sister] | 43 (13.7) |
|  | Extended Family Member | 3 (0.9) |
| Ever had an episode of opportunistic infection (OI) | No | 71 (18.5) |
|  | Yes | 313 (81.5) |
| Types of OI ever had [*multiple responses possible] (n= 313) | Mouth and throat problems (Oral thrush/candidiasis, Mouth ulcer, Tonsilitis) | 28 (8.9) |
|  | Chronic Diarrheal Disease | 50 (16.0) |
|  | Chronic Cough (Cold, Pneumonia, Tuberculosis) | 180 (57.5) |
|  | Herpes Zoster | 12 (3.8) |
|  | Other Infection | 69 (22.0) |
| Ever had any other infection [*multiple response possible] (n=69) | Accessary organ problems (liver disease) | 4 (5.8) |
|  | Central Nervous System problem | 23 (33.3) |
|  | Cardiovascular system Problem | 9 (13.0) |
|  | Dermatologic system problem | 32 (46.4) |
|  | Ear, Eye, Nose, and Throat (EENT) problems | 9 (13.0) |
|  | Haematologic system problem | 13 (18.8) |
|  | Gastrointestinal system problem | 39 (56.5) |
|  | Respiratory system problem | 1 (1.4) |
|  | Urinary system problem | 11 (15.9) |
|  | Endocrine and musculoskeletal system problems | 4 (5.8) |
| Ever had a long-standing disease such as Tuberculosis (yes) | | 127 (33.1) |
| Had any other illness in the last 3 months (yes) | | 192 (50.0) |
| Types of illness had in the last 3 months (*multiple responses possible) (n=192) | Accessary organ problem (liver disease) | 3 (1.6) |
|  | Central nervous system problem | 23 (12.0) |
|  | Dermatologic system problem | 10 (5.2) |
|  | Ear, Eye, Nose, and Throat (EENT) problems | 15 (7.8) |
|  | Haematologic problem | 4 (2.1) |
|  | Gastrointestinal system problem | 37 (19.3) |
|  | Respiratory system problem | 129 (67.2) |
|  | Urinary system problem | 6 (3.1) |
|  | Other system problems (e.g. Musculo-skeletal and reproductive) | 2 (1.0) |
| Time when ART initiated | < 15 years ago | 142 (37.0) |
|  | ≥15 years ago | 242 (63.0) |
| Ever missed an ART clinic appointment for an ART follow-up (yes) | | 182 (47.4) |
