# Supplementary material for: Clinical, lifestyle, environmental and dietary determinants of malnutrition in adolescents on antiretroviral therapy in Ethiopia
Source: PLOS Glob Public Health. 2026 Jun 26;6(6):e0005003. doi: 10.1371/journal.pgph.0005003 (PMC13309033; doi:10.1371/journal.pgph.0005003)
Supplement: S2 Table — (DOCX) [file pgph.0005003.s004.docx]

**Supporting Information**

**S2 Table. Medical record profile of adolescents living with HIV on ART follow-up in Ethiopia, 2024 (n=384)**

| Variables | Description | Frequency N (%) |
| --- | --- | --- |
| Age at diagnosis and ART enrolment | < 4 years | 190 (49.5) |
|  | ≥ 4 years | 194 (50.5) |
| Mean ± SD | 4.66 ± 3.7 years |  |
| Weight during ART enrolment | < 14 kg | 189 (49.2) |
|  | ≥ 14 kg | 195 (50.8) |
| Height during ART enrolment | < 104 cm | 190 (49.5) |
|  | ≥ 104 cm | 194 (50.5) |
| CD_4_ status on ART enrolment | < 200 cell/mm3 | 25 (6.5) |
|  | 200 – 500 cell/mm3 | 11 (30.2) |
|  | 500 – 1500 cells/mm3 | 209 (54.4) |
|  | ≥1500 cell/mm3 | 23 (6.0) |
|  | Unknown | 11 (2.9) |
| CD_4_ status within the last 3 months | 200 – 500 cell/mm3 | 2 (0.5) |
|  | 500 – 1500 cells/mm3 | 4 (1.0) |
|  | ≥1500 cell/mm3 | 4 (1.0) |
|  | Unknown | 374 (97.4) |
| Viral load status within the last 3 months | ≥ 150 copies per ml of blood | 4 (1.0) |
|  | < 150 copies per ml of blood | 375 (97.7) |
|  | Unknown | 5 (1.3) |
| Haemoglobin level (n=359) | Low levels (< 11.5 gm/dl) | 55 (15.3) |
|  | Normal level (11.5 – 15.0 gm/dl) | 284 (79.1) |
|  | High level (≥ 15 gm/dl) | 20 (5.6) |
| Alanine Transaminase (ALT or SGPT) (n=234) | Normal level (0 – 50 U/L) | 221 (94.4) |
|  | Elevated level (> 50 U/L) | 13 (5.6) |
| Aspartate Amino Transferase (AST or SGOT) (n=236) | Normal Level (0 – 60 U/L) | 227 (96.2) |
|  | Elevated Level (> 60 U/L) | 9 (3.8) |
| WHO Clinical stage before ART initiation | Stage I | 247 (64.3) |
|  | Stage II | 2 (0.5) |
|  | Stage III | 113 (29.4) |
|  | Stage IV | 22 (5.7) |
| Current WHO clinical stage | Stage I | 371 (96.6) |
|  | Stage II | 7 (1.8) |
|  | Stage III | 6 (1.6) |
| *Note: ALT- alanine aminotransferase; AST - aspartate aminotransferase; ART- Anti-Retroviral Therapy; SD-Standard Deviation; SGPT- serum glutamic-pyruvic transaminase; SGOT - serum glutamic-oxaloacetic transaminase; WHO-world Health Organization* | | |
