# Supplementary material for: Clinical, lifestyle, environmental and dietary determinants of malnutrition in adolescents on antiretroviral therapy in Ethiopia
Source: PLOS Glob Public Health. 2026 Jun 26;6(6):e0005003. doi: 10.1371/journal.pgph.0005003 (PMC13309033; doi:10.1371/journal.pgph.0005003)
Supplement: S3 Table — (DOCX) [file pgph.0005003.s005.docx]

**Supporting Information**

**S3 Table. Environmental and dietary factors: household food availability, supplementation and dietary intakes of adolescents living with HIV on ART follow-up in Ethiopia, 2024 (n=384)**

| Variables | Description | Frequency N (%) |
| --- | --- | --- |
| Ever worried about not having enough food in the household (yes) | | 132 (34.4) |
| Ever unable to eat preferred food due to resource limitation (yes) | | 208 (54.2) |
| Ever eaten a limited variety of food due to resource limitation (yes) | | 204 (53.1) |
| Ever eaten some foods did not want to eat due to resource limitations to obtain other types of food (yes) | | 154 (40.1) |
| Ever eaten smaller meals than needed because not enough food available (yes) | | 154 (40.1) |
| Ever eaten fewer meals in a day because there was not enough food (yes) | | 172 (44.8) |
| Ever had no kind of food to eat due to limited resource (yes) | | 99 (25.8) |
| Ever gone to bed/sleep at night hungry because there was not enough food at home (yes) | | 213 (55.3) |
| Ever gone day and night without eating because there is not enough food (yes) | | 59 (15.4) |
| Usual meals frequency in 24 hours (day and night) | ≤ 2 meals | 60 (15.6) |
|  | 3 – 5 meals | 324 (84.4) |
| Have you ever skipped a meal because food was not available (yes) | | 321 (83.6) |
| Ever had feeding-related complications after eating food, such as vomiting, diarrhea, loss of appetite (yes) | | 110 (28.6) |
| Ever been provided with nutrition supplements (yes) | | 238 (62.0) |
| Type of supplementation ever taken (n=238) | Ready to use Therapeutic Feeding (RUTF) | 238 (100) |
| Number of supplementations taken per day (n=238) | 1 sachet RUTF per a day | 153 (64.3) |
|  | 1.5 – 2 sachet RUTF per day | 57 (23.9) |
|  | 3 – 4 sachet RUTF per day | 28 (11.8) |
| Duration supplementation taken (n=238) | ≤ 2 months | 151 (63.4) |
|  | 3 – 4 months | 74(31.1) |
|  | 5 or more months | 13 (5.5) |
| Place where supplementation taken (n=238) | ART Centre of the hospital | 236 (99.2) |
|  | NGO | 2 (0.8) |

*Note: ART- Anti-Retroviral Therapy; RUTF- Ready to use Therapeutic Feeding; NGO- Non-Government Organization*;
