# Supplementary material for: Clinical, lifestyle, environmental and dietary determinants of malnutrition in adolescents on antiretroviral therapy in Ethiopia
Source: PLOS Glob Public Health. 2026 Jun 26;6(6):e0005003. doi: 10.1371/journal.pgph.0005003 (PMC13309033; doi:10.1371/journal.pgph.0005003)
Supplement: S4 Table — (DOCX) [file pgph.0005003.s006.docx]

**Supporting Information**

**S4 Table. Environmental factors: household water and toilet facility-related characteristics of adolescents living with HIV on ART follow-up in Ethiopia, 2024 (n=384)**

| Variables | Description | Frequency N (%) |
| --- | --- | --- |
| Live in households with a clean water supply (yes) | | 297 (77.3) |
| Treat the water to make it safe to drink or for other purpose (n=87) | No | 44 (50.6) |
|  | Yes | 42 (48.3) |
|  | Unknown | 1 (1.1) |
| Methods used for making the water safe for drinking [*multiple responses possible] (n=42) | |  |
| Use wuha agar (chlorine-based water treatment solution) | | 26 (61.9) |
| Use a water filter (ceramic/sand/composite) | | 23 (54.8) |
| Boil the water | | 14 (33.3) |
| Let the water stand and settle (sedimentation) | | 14 (33.3) |
| Use of highland mineral water | | 5 (11.9) |
| Strain through a cloth | | 3 (7.1) |
| Solar disinfection | | 1 (2.4) |
| Toilet facilities of the households | Flush or pour-flush toilet | 66 (17.2) |
|  | Pit latrine | 315 (82.0) |
|  | Communal Latrine | 2 (0.5) |
|  | Open field | 1 (0.3) |
