# Supplementary material for: Clinical, lifestyle, environmental and dietary determinants of malnutrition in adolescents on antiretroviral therapy in Ethiopia
Source: PLOS Glob Public Health. 2026 Jun 26;6(6):e0005003. doi: 10.1371/journal.pgph.0005003 (PMC13309033; doi:10.1371/journal.pgph.0005003)
Supplement: S5 Table — (DOCX) [file pgph.0005003.s007.docx]

**Supporting Information**

| **S5 Table. Correlation Analysis to determine variables to include in modelling for thinness** | | | | | | | | | | | | | |
| --- | --- | --- | --- | --- | --- | --- | --- | --- | --- | --- | --- | --- | --- |
|  | | | | | | | | | | | | | |
|  | | BMI-for-Age (BAZ) | Sex /Gender | Age | Food Insecurity concern | HH Monthly Income | Ever had a nutritional assessment during ART initiation | Length of time aware of HIV status | Ever had long-lasting infections such as tuberculosis | GAD (anxiety) | PHQ-9 (depression) | Ever taken nutritional supplements |  |
| BMI-for-Age (BAZ) | Pearson Correlation | 1 | -.270^**^ | -.074 | -.105^*^ | -.065 | -.083 | .081 | -.132^**^ | -.121^*^ | -.114^*^ | -.142^**^ |  |
|  | Sig. (2-tailed) |  | .000 | .149 | .039 | .203 | .103 | .112 | .009 | .017 | .026 | .005 |  |
|  | Sum of Squares and Cross-products | 70.477 | -22.133 | -7.617 | -8.648 | -5.313 | -6.156 | 5.352 | -10.242 | -9.984 | -4.703 | -11.359 |  |
|  | Covariance | .184 | -.058 | -.020 | -.023 | -.014 | -.016 | .014 | -.027 | -.026 | -.012 | -.030 |  |
|  | N | 384 | 384 | 384 | 384 | 384 | 384 | 384 | 384 | 384 | 384 | 384 |  |
| Sex /Gender | Pearson Correlation | -.270^**^ | 1 | .019 | .152^**^ | .016 | .021 | -.059 | -.028 | -.069 | .021 | .132^**^ |  |
|  | Sig. (2-tailed) | .000 |  | .704 | .003 | .748 | .686 | .250 | .582 | .179 | .680 | .009 |  |
|  | Sum of Squares and Cross-products | -22.133 | 95.414 | 2.336 | 14.492 | 1.563 | 1.781 | -4.508 | -2.539 | -6.578 | 1.016 | 12.297 |  |
|  | Covariance | -.058 | .249 | .006 | .038 | .004 | .005 | -.012 | -.007 | -.017 | .003 | .032 |  |
|  | N | 384 | 384 | 384 | 384 | 384 | 384 | 384 | 384 | 384 | 384 | 384 |  |
| Age | Pearson Correlation | -.074 | .019 | 1 | -.082 | .137^**^ | .020 | .326^**^ | .022 | .114^*^ | -.003 | -.010 |  |
|  | Sig. (2-tailed) | .149 | .704 |  | .107 | .007 | .689 | .000 | .672 | .025 | .953 | .850 |  |
|  | Sum of Squares and Cross-products | -7.617 | 2.336 | 151.247 | -9.909 | 16.438 | 2.219 | 31.424 | 2.456 | 13.745 | -.182 | -1.130 |  |
|  | Covariance | -.020 | .006 | .395 | -.026 | .043 | .006 | .082 | .006 | .036 | .000 | -.003 |  |
|  | N | 384 | 384 | 384 | 384 | 384 | 384 | 384 | 384 | 384 | 384 | 384 |  |
| Food Insecurity concern | Pearson Correlation | -.105^*^ | .152^**^ | -.082 | 1 | -.151^**^ | .190^**^ | -.080 | -.036 | .276^**^ | .122^*^ | .183^**^ |  |
|  | Sig. (2-tailed) | .039 | .003 | .107 |  | .003 | .000 | .119 | .488 | .000 | .017 | .000 |  |
|  | Sum of Squares and Cross-products | -8.648 | 14.492 | -9.909 | 95.560 | -14.313 | 16.344 | -6.107 | -3.201 | 26.432 | 5.880 | 17.057 |  |
|  | Covariance | -.023 | .038 | -.026 | .250 | -.037 | .043 | -.016 | -.008 | .069 | .015 | .045 |  |
|  | N | 384 | 384 | 384 | 384 | 384 | 384 | 384 | 384 | 384 | 384 | 384 |  |
| HH Monthly Income | Pearson Correlation | -.065 | .016 | .137^**^ | -.151^**^ | 1 | -.114^*^ | .114^*^ | -.073 | .030 | .076 | -.055 |  |
|  | Sig. (2-tailed) | .203 | .748 | .007 | .003 |  | .026 | .026 | .152 | .555 | .138 | .279 |  |
|  | Sum of Squares and Cross-products | -5.313 | 1.563 | 16.438 | -14.313 | 94.500 | -9.750 | 8.688 | -6.563 | 2.875 | 3.625 | -5.125 |  |
|  | Covariance | -.014 | .004 | .043 | -.037 | .247 | -.025 | .023 | -.017 | .008 | .009 | -.013 |  |
|  | N | 384 | 384 | 384 | 384 | 384 | 384 | 384 | 384 | 384 | 384 | 384 |  |
| Ever had a nutritional assessment during ART initiation | Pearson Correlation | -.083 | .021 | .020 | .190^**^ | -.114^*^ | 1 | -.067 | .132^**^ | .040 | .053 | .023 |  |
|  | Sig. (2-tailed) | .103 | .686 | .689 | .000 | .026 |  | .188 | .010 | .436 | .297 | .652 |  |
|  | Sum of Squares and Cross-products | -6.156 | 1.781 | 2.219 | 16.344 | -9.750 | 77.625 | -4.656 | 10.719 | 3.438 | 2.313 | 1.938 |  |
|  | Covariance | -.016 | .005 | .006 | .043 | -.025 | .203 | -.012 | .028 | .009 | .006 | .005 |  |
|  | N | 384 | 384 | 384 | 384 | 384 | 384 | 384 | 384 | 384 | 384 | 384 |  |
| Length of time aware of HIV status | Pearson Correlation | .081 | -.059 | .326^**^ | -.080 | .114^*^ | -.067 | 1 | -.090 | .066 | -.020 | .050 |  |
|  | Sig. (2-tailed) | .112 | .250 | .000 | .119 | .026 | .188 |  | .077 | .195 | .691 | .329 |  |
|  | Sum of Squares and Cross-products | 5.352 | -4.508 | 31.424 | -6.107 | 8.688 | -4.656 | 61.560 | -6.534 | 5.099 | -.786 | 3.724 |  |
|  | Covariance | .014 | -.012 | .082 | -.016 | .023 | -.012 | .161 | -.017 | .013 | -.002 | .010 |  |
|  | N | 384 | 384 | 384 | 384 | 384 | 384 | 384 | 384 | 384 | 384 | 384 |  |
| Ever had a long-standing infection such as Tuberculosis | Pearson Correlation | -.132^**^ | -.028 | .022 | -.036 | -.073 | .132^**^ | -.090 | 1 | .146^**^ | .031 | -.020 |  |
|  | Sig. (2-tailed) | .009 | .582 | .672 | .488 | .152 | .010 | .077 |  | .004 | .546 | .703 |  |
|  | Sum of Squares and Cross-products | -10.242 | -2.539 | 2.456 | -3.201 | -6.563 | 10.719 | -6.534 | 84.997 | 13.161 | 1.401 | -1.714 |  |
|  | Covariance | -.027 | -.007 | .006 | -.008 | -.017 | .028 | -.017 | .222 | .034 | .004 | -.004 |  |
|  | N | 384 | 384 | 384 | 384 | 384 | 384 | 384 | 384 | 384 | 384 | 384 |  |
| GAD (anxiety) | Pearson Correlation | -.121^*^ | -.069 | .114^*^ | .276^**^ | .030 | .040 | .066 | .146^**^ | 1 | .272^**^ | .003 |  |
|  | Sig. (2-tailed) | .017 | .179 | .025 | .000 | .555 | .436 | .195 | .004 |  | .000 | .960 |  |
|  | Sum of Squares and Cross-products | -9.984 | -6.578 | 13.745 | 26.432 | 2.875 | 3.438 | 5.099 | 13.161 | 95.990 | 13.135 | .240 |  |
|  | Covariance | -.026 | -.017 | .036 | .069 | .008 | .009 | .013 | .034 | .251 | .034 | .001 |  |
|  | N | 384 | 384 | 384 | 384 | 384 | 384 | 384 | 384 | 384 | 384 | 384 |  |
| PHQ-9 (depression) | Pearson Correlation | -.114^*^ | .021 | -.003 | .122^*^ | .076 | .053 | -.020 | .031 | .272^**^ | 1 | .019 |  |
|  | Sig. (2-tailed) | .026 | .680 | .953 | .017 | .138 | .297 | .691 | .546 | .000 |  | .712 |  |
|  | Sum of Squares and Cross-products | -4.703 | 1.016 | -.182 | 5.880 | 3.625 | 2.313 | -.786 | 1.401 | 13.135 | 24.240 | .885 |  |
|  | Covariance | -.012 | .003 | .000 | .015 | .009 | .006 | -.002 | .004 | .034 | .063 | .002 |  |
|  | N | 384 | 384 | 384 | 384 | 384 | 384 | 384 | 384 | 384 | 384 | 384 |  |
| Ever taken nutritional supplements | Pearson Correlation | -.142^**^ | .132^**^ | -.010 | .183^**^ | -.055 | .023 | .050 | -.020 | .003 | .019 | 1 |  |
|  | Sig. (2-tailed) | .005 | .009 | .850 | .000 | .279 | .652 | .329 | .703 | .960 | .712 |  |  |
|  | Sum of Squares and Cross-products | -11.359 | 12.297 | -1.130 | 17.057 | -5.125 | 1.938 | 3.724 | -1.714 | .240 | .885 | 90.490 |  |
|  | Covariance | -.030 | .032 | -.003 | .045 | -.013 | .005 | .010 | -.004 | .001 | .002 | .236 |  |
|  | N | 384 | 384 | 384 | 384 | 384 | 384 | 384 | 384 | 384 | 384 | 384 |  |
| ***Note:*** *ART-Anti-Retroviral Therapy; GAD- Generalized Anxiety Disorder; PHQ-9 – Patient Health Questionnaire (9 item). HH- Household; HIV- Human Immunodeficiency Virus; BAZ- Body Mass Index- for-Age Z-score*. **#** Pearson Correlation coefficients reported  **. Correlation is significant at the 0.01 level (2-tailed). | | | | | | | | | | | | | |
