# Supplementary material for: Clinical, lifestyle, environmental and dietary determinants of malnutrition in adolescents on antiretroviral therapy in Ethiopia
Source: PLOS Glob Public Health. 2026 Jun 26;6(6):e0005003. doi: 10.1371/journal.pgph.0005003 (PMC13309033; doi:10.1371/journal.pgph.0005003)
Supplement: S6 Table — (DOCX) [file pgph.0005003.s008.docx]

**Supporting Information**

| **S6 Table. Correlation Analysis to determine variables to include in modelling for acute malnutrition** | | | | | | | | | | | | | | | |
| --- | --- | --- | --- | --- | --- | --- | --- | --- | --- | --- | --- | --- | --- | --- | --- |
|  | | Sex | ALT (SGPT) | Hgb | GAD | PHQ-9 | Food Insecurity concern | Nutritional supplements | Long-standing infection such as Tuberculosis | Length of time aware of HIV status | HH Monthly Income | Family Size | Age | Number of meals in 24 hours period | MUAC |
| Sex | Pearson Correlation | 1 | .015 | .017 | -.069 | -.083 | .152^**^ | .132^**^ | -.028 | -.059 | .016 | .010 | -.024 | -.019 | -.125^*^ |
|  | Sig. (2-tailed) |  | .825 | .745 | .179 | .106 | .003 | .009 | .582 | .250 | .748 | .842 | .641 | .706 | .014 |
|  | Sum of Squares and Cross-products | 95.414 | .389 | 1.379 | -6.578 | -7.891 | 14.492 | 12.297 | -2.539 | -4.508 | 1.563 | .922 | -2.016 | -1.344 | -11.898 |
|  | Covariance | .249 | .002 | .004 | -.017 | -.021 | .038 | .032 | -.007 | -.012 | .004 | .002 | -.005 | -.004 | -.031 |
|  | N | 384 | 234 | 359 | 384 | 384 | 384 | 384 | 384 | 384 | 384 | 384 | 384 | 384 | 384 |
| ALT(SGPT) | Pearson Correlation | .015 | 1 | -.174^**^ | .012 | .019 | -.010 | .029 | .023 | .019 | .065 | -.022 | .150^*^ | .073 | -.090 |
|  | Sig. (2-tailed) | .825 |  | .009 | .850 | .776 | .875 | .657 | .725 | .775 | .319 | .740 | .021 | .266 | .168 |
|  | Sum of Squares and Cross-products | .389 | 12.278 | -4.026 | .333 | .500 | -.278 | .778 | .611 | .444 | 1.722 | -.556 | 3.556 | 1.556 | -2.389 |
|  | Covariance | .002 | .053 | -.018 | .001 | .002 | -.001 | .003 | .003 | .002 | .007 | -.002 | .015 | .007 | -.010 |
|  | N | 234 | 234 | 227 | 234 | 234 | 234 | 234 | 234 | 234 | 234 | 234 | 234 | 234 | 234 |
| Hb | Pearson Correlation | .017 | -.174^**^ | 1 | .006 | -.021 | -.062 | -.014 | -.146^**^ | .085 | .068 | -.034 | -.008 | .124^*^ | .116^*^ |
|  | Sig. (2-tailed) | .745 | .009 |  | .915 | .685 | .245 | .796 | .005 | .106 | .199 | .515 | .885 | .018 | .028 |
|  | Sum of Squares and Cross-products | 1.379 | -4.026 | 71.588 | .451 | -1.719 | -4.914 | -1.064 | -11.106 | 5.591 | 5.404 | -2.602 | -.543 | 7.345 | 9.209 |
|  | Covariance | .004 | -.018 | .200 | .001 | -.005 | -.014 | -.003 | -.031 | .016 | .015 | -.007 | -.002 | .021 | .026 |
|  | N | 359 | 227 | 359 | 359 | 359 | 359 | 359 | 359 | 359 | 359 | 359 | 359 | 359 | 359 |
| GAD | Pearson Correlation | -.069 | .012 | .006 | 1 | .521^**^ | .276^**^ | .003 | .146^**^ | .066 | .030 | -.051 | .077 | -.119^*^ | -.038 |
|  | Sig. (2-tailed) | .179 | .850 | .915 |  | .000 | .000 | .960 | .004 | .195 | .555 | .314 | .132 | .019 | .458 |
|  | Sum of Squares and Cross-products | -6.578 | .333 | .451 | 95.990 | 49.948 | 26.432 | .240 | 13.161 | 5.099 | 2.875 | -4.677 | 6.531 | -8.313 | -3.620 |
|  | Covariance | -.017 | .001 | .001 | .251 | .130 | .069 | .001 | .034 | .013 | .008 | -.012 | .017 | -.022 | -.009 |
|  | N | 384 | 234 | 359 | 384 | 384 | 384 | 384 | 384 | 384 | 384 | 384 | 384 | 384 | 384 |
| PHQ-9 | Pearson Correlation | -.083 | .019 | -.021 | .521^**^ | 1 | .274^**^ | .045 | .098 | .098 | -.038 | -.026 | .020 | -.080 | -.064 |
|  | Sig. (2-tailed) | .106 | .776 | .685 | .000 |  | .000 | .378 | .056 | .056 | .456 | .608 | .702 | .118 | .210 |
|  | Sum of Squares and Cross-products | -7.891 | .500 | -1.719 | 49.948 | 95.740 | 26.161 | 4.198 | 8.807 | 7.495 | -3.625 | -2.385 | 1.656 | -5.563 | -6.099 |
|  | Covariance | -.021 | .002 | -.005 | .130 | .250 | .068 | .011 | .023 | .020 | -.009 | -.006 | .004 | -.015 | -.016 |
|  | N | 384 | 234 | 359 | 384 | 384 | 384 | 384 | 384 | 384 | 384 | 384 | 384 | 384 | 384 |
| Recode Food Insecurity concern | Pearson Correlation | .152^**^ | -.010 | -.062 | .276^**^ | .274^**^ | 1 | .183^**^ | -.036 | -.080 | -.151^**^ | -.071 | -.018 | -.230^**^ | -.145^**^ |
|  | Sig. (2-tailed) | .003 | .875 | .245 | .000 | .000 |  | .000 | .488 | .119 | .003 | .167 | .721 | .000 | .004 |
|  | Sum of Squares and Cross-products | 14.492 | -.278 | -4.914 | 26.432 | 26.161 | 95.560 | 17.057 | -3.201 | -6.107 | -14.313 | -6.401 | -1.547 | -16.031 | -13.779 |
|  | Covariance | .038 | -.001 | -.014 | .069 | .068 | .250 | .045 | -.008 | -.016 | -.037 | -.017 | -.004 | -.042 | -.036 |
|  | N | 384 | 234 | 359 | 384 | 384 | 384 | 384 | 384 | 384 | 384 | 384 | 384 | 384 | 384 |
| Ever taken nutritional supplements | Pearson Correlation | .132^**^ | .029 | -.014 | .003 | .045 | .183^**^ | 1 | -.020 | .050 | -.055 | -.062 | -.027 | .018 | -.105^*^ |
|  | Sig. (2-tailed) | .009 | .657 | .796 | .960 | .378 | .000 |  | .703 | .329 | .279 | .229 | .599 | .732 | .039 |
|  | Sum of Squares and Cross-products | 12.297 | .778 | -1.064 | .240 | 4.198 | 17.057 | 90.490 | -1.714 | 3.724 | -5.125 | -5.427 | -2.219 | 1.188 | -9.745 |
|  | Covariance | .032 | .003 | -.003 | .001 | .011 | .045 | .236 | -.004 | .010 | -.013 | -.014 | -.006 | .003 | -.025 |
|  | N | 384 | 234 | 359 | 384 | 384 | 384 | 384 | 384 | 384 | 384 | 384 | 384 | 384 | 384 |
| Ever had a long-standing infection such as Tuberculosis? | Pearson Correlation | -.028 | .023 | -.146^**^ | .146^**^ | .098 | -.036 | -.020 | 1 | -.090 | -.073 | .035 | .053 | -.033 | -.110^*^ |
|  | Sig. (2-tailed) | .582 | .725 | .005 | .004 | .056 | .488 | .703 |  | .077 | .152 | .494 | .296 | .521 | .031 |
|  | Sum of Squares and Cross-products | -2.539 | .611 | -11.106 | 13.161 | 8.807 | -3.201 | -1.714 | 84.997 | -6.534 | -6.563 | 2.995 | 4.266 | -2.156 | -9.893 |
|  | Covariance | -.007 | .003 | -.031 | .034 | .023 | -.008 | -.004 | .222 | -.017 | -.017 | .008 | .011 | -.006 | -.026 |
|  | N | 384 | 234 | 359 | 384 | 384 | 384 | 384 | 384 | 384 | 384 | 384 | 384 | 384 | 384 |
| Length of time aware of HIV status | Pearson Correlation | -.059 | .019 | .085 | .066 | .098 | -.080 | .050 | -.090 | 1 | .114^*^ | -.042 | .242^**^ | .125^*^ | .300^**^ |
|  | Sig. (2-tailed) | .250 | .775 | .106 | .195 | .056 | .119 | .329 | .077 |  | .026 | .410 | .000 | .014 | .000 |
|  | Sum of Squares and Cross-products | -4.508 | .444 | 5.591 | 5.099 | 7.495 | -6.107 | 3.724 | -6.534 | 61.560 | 8.688 | -3.068 | 16.453 | 6.969 | 22.888 |
|  | Covariance | -.012 | .002 | .016 | .013 | .020 | -.016 | .010 | -.017 | .161 | .023 | -.008 | .043 | .018 | .060 |
|  | N | 384 | 234 | 359 | 384 | 384 | 384 | 384 | 384 | 384 | 384 | 384 | 384 | 384 | 384 |
| HH Monthly Income | Pearson Correlation | .016 | .065 | .068 | .030 | -.038 | -.151^**^ | -.055 | -.073 | .114^*^ | 1 | .121^*^ | .064 | .105^*^ | .106^*^ |
|  | Sig. (2-tailed) | .748 | .319 | .199 | .555 | .456 | .003 | .279 | .152 | .026 |  | .018 | .212 | .040 | .037 |
|  | Sum of Squares and Cross-products | 1.563 | 1.722 | 5.404 | 2.875 | -3.625 | -14.313 | -5.125 | -6.563 | 8.688 | 94.500 | 10.875 | 5.375 | 7.250 | 10.063 |
|  | Covariance | .004 | .007 | .015 | .008 | -.009 | -.037 | -.013 | -.017 | .023 | .247 | .028 | .014 | .019 | .026 |
|  | N | 384 | 234 | 359 | 384 | 384 | 384 | 384 | 384 | 384 | 384 | 384 | 384 | 384 | 384 |
| Family Size | Pearson Correlation | .010 | -.022 | -.034 | -.051 | -.026 | -.071 | -.062 | .035 | -.042 | .121^*^ | 1 | -.006 | -.050 | .013 |
|  | Sig. (2-tailed) | .842 | .740 | .515 | .314 | .608 | .167 | .229 | .494 | .410 | .018 |  | .909 | .326 | .793 |
|  | Sum of Squares and Cross-products | .922 | -.556 | -2.602 | -4.677 | -2.385 | -6.401 | -5.427 | 2.995 | -3.068 | 10.875 | 85.990 | -.469 | -3.313 | 1.214 |
|  | Covariance | .002 | -.002 | -.007 | -.012 | -.006 | -.017 | -.014 | .008 | -.008 | .028 | .225 | -.001 | -.009 | .003 |
|  | N | 384 | 234 | 359 | 384 | 384 | 384 | 384 | 384 | 384 | 384 | 384 | 384 | 384 | 384 |
| Age | Pearson Correlation | -.024 | .150^*^ | -.008 | .077 | .020 | -.018 | -.027 | .053 | .242^**^ | .064 | -.006 | 1 | .048 | .274^**^ |
|  | Sig. (2-tailed) | .641 | .021 | .885 | .132 | .702 | .721 | .599 | .296 | .000 | .212 | .909 |  | .351 | .000 |
|  | Sum of Squares and Cross-products | -2.016 | 3.556 | -.543 | 6.531 | 1.656 | -1.547 | -2.219 | 4.266 | 16.453 | 5.375 | -.469 | 74.906 | 2.938 | 23.109 |
|  | Covariance | -.005 | .015 | -.002 | .017 | .004 | -.004 | -.006 | .011 | .043 | .014 | -.001 | .196 | .008 | .060 |
|  | N | 384 | 234 | 359 | 384 | 384 | 384 | 384 | 384 | 384 | 384 | 384 | 384 | 384 | 384 |
| Number of meals in 24 hours period | Pearson Correlation | -.019 | .073 | .124^*^ | -.119^*^ | -.080 | -.230^**^ | .018 | -.033 | .125^*^ | .105^*^ | -.050 | .048 | 1 | .093 |
|  | Sig. (2-tailed) | .706 | .266 | .018 | .019 | .118 | .000 | .732 | .521 | .014 | .040 | .326 | .351 |  | .070 |
|  | Sum of Squares and Cross-products | -1.344 | 1.556 | 7.345 | -8.313 | -5.563 | -16.031 | 1.188 | -2.156 | 6.969 | 7.250 | -3.313 | 2.938 | 50.625 | 6.406 |
|  | Covariance | -.004 | .007 | .021 | -.022 | -.015 | -.042 | .003 | -.006 | .018 | .019 | -.009 | .008 | .132 | .017 |
|  | N | 384 | 234 | 359 | 384 | 384 | 384 | 384 | 384 | 384 | 384 | 384 | 384 | 384 | 384 |
| MUAC | Pearson Correlation | -.125^*^ | -.090 | .116^*^ | -.038 | -.064 | -.145^**^ | -.105^*^ | -.110^*^ | .300^**^ | .106^*^ | .013 | .274^**^ | .093 | 1 |
|  | Sig. (2-tailed) | .014 | .168 | .028 | .458 | .210 | .004 | .039 | .031 | .000 | .037 | .793 | .000 | .070 |  |
|  | Sum of Squares and Cross-products | -11.898 | -2.389 | 9.209 | -3.620 | -6.099 | -13.779 | -9.745 | -9.893 | 22.888 | 10.063 | 1.214 | 23.109 | 6.406 | 94.622 |
|  | Covariance | -.031 | -.010 | .026 | -.009 | -.016 | -.036 | -.025 | -.026 | .060 | .026 | .003 | .060 | .017 | .247 |
|  | N | 384 | 234 | 359 | 384 | 384 | 384 | 384 | 384 | 384 | 384 | 384 | 384 | 384 | 384 |
| ***Note:*** *ALT- alanine aminotransferase; ART-Anti-Retroviral Therapy; GAD- Generalized Anxiety Disorder; Hb- Haemoglobin; HH- Household; HIV- Human Immunodeficiency Virus; BAZ- Body Mass Index- for-Age Z-score; MUAC- Mid-Upper Arm Circumference; SGPT- serum glutamic-pyruvic transaminase; GAD – Generalised Anxiety Disorder; PHQ-9 – Patient Health Questionnaire (9 item)*.  # Pearson Correlation coefficients reported  **. Correlation is significant at the 0.01 level (2-tailed). | | | | | | | | | | | | | | | |
| *. Correlation is significant at the 0.05 level (2-tailed). | | | | | | | | | | | | | | | |
