# Supplementary material for: Clinical, lifestyle, environmental and dietary determinants of malnutrition in adolescents on antiretroviral therapy in Ethiopia
Source: PLOS Glob Public Health. 2026 Jun 26;6(6):e0005003. doi: 10.1371/journal.pgph.0005003 (PMC13309033; doi:10.1371/journal.pgph.0005003)
Supplement: Attachment — Submitted filename: Response to Editor and Reviewers questions.docx [file pgph.0005003.s010.docx]

**Response to Editor and Reviewers**

**Manuscript ID:** PGPH-D-25-01939
**Title:** ***Clinical, Lifestyle, Environmental and Dietary Factors associated with Malnutrition in Adolescents on Antiretroviral Therapy in Ethiopia***
**Corresponding Author:** Dr. Meless Gebrie Bore

We sincerely thank the Academic Editor and both reviewers for their constructive feedback, which has greatly strengthened the clarity, rigor, and presentation of our manuscript.
All comments were carefully addressed, and corresponding revisions have been made in the manuscript (tracked in the version labeled *“Revised Manuscript with Track Changes”*).
Below, we provide a point-by-point response.

**Response to Editor’s Comments**

**Comment 1:**

Methods: It is unclear whether participants were recruited from inpatient or outpatient settings; the description of “survey design” lacks precision; details on sample size and data collection are overly long and could be streamlined; and the decision to recode qualitative data into quantitative variables is unconventional and should be justified.

**Response:**
We appreciate this observation. We have clarified that participants were **recruited from outpatient ART clinics** at the eight hospitals included in the study (section 2.1).

The term “survey design” has been expanded to ‘An institutional-based mixed-methods design was employed. The predominant quantitative component used a cross-sectional survey design, supplemented by clinical assessments and extraction of clinical record data. The survey consisted of a composite structured, interviewer-administered questionnaire.” (section 2.2).

Redundant details in the sample size and data collection subsections have been shortened for clarity to “The sample size was calculated to ensure adequate power for analyses of nutritional status, associated factors, and dietary patterns among adolescents living with HIV (ALHIV) on ART. Based on published prevalence estimates and power calculations for key variables [16, 17], the largest required sample size was 384, which was adopted as the final sample size for all analyses” (section 2.3).

We also added justification for integrating qualitative data into the quantitative dataset, explaining that this was done to quantify participant-reported challenges related to nutrition, food security, and adherence for inclusion in regression analysis (section 2.5, paragraph 3). The changed statement is as follows: “Qualitative data were initially categorized to facilitate integration with the quantitative dataset and complement statistical findings [34-36]. Additionally, a thematic analysis was conducted, where two independent researchers inductively coded qualitative responses to identify key themes. Discrepancies were resolved by consensus, and representative illustrative quotes were selected to enrich the interpretation of findings. Each participant and healthcare provider were assigned an identifier to prevent duplication and ensure full de-identification. All analytical procedures adhered to the ethical standards and approvals outlined in the declarations section.”

**Comment 2:**

Results: Some sentences (e.g., age at ART initiation) are unclear; the repeated use of “determinant factors” implies causality; and certain associations may be bidirectional or confounded.

**Response:**
We revised the Results section for clarity, especially the statement on **age at ART initiation**, which now reads: “The median age at HIV diagnosis and ART initiation was 3.95 years (mean 4.66 ± 3.7 years). Half of the participants (50.5%, n=194) began ART before the age of 4” (section 3.1.3).

We have replaced “determinant factors” with “associated factors” throughout the manuscript (section 3.2.1 and 3.2.2) and carefully rephrased causal or bidirectional relationships (e.g., TB–malnutrition, anemia–malnutrition, anxiety–thinness) to emphasize correlation rather than causation (Section 3.2.1 and 3.2.2: Clinical variable)

**Comment 3:**

Discussion: Interpretations sometimes overstate directionality; translating qualitative responses into quantitative variables limited insights; more thematic coding and illustrative quotes could have strengthened the findings.

**Response:**
We have revised the Discussion to be more cautious of directionality, remove statements of causality (section 4, throughout)

and highlight the cross-sectional design’s limitations (section 4.1).

Additionally, we expanded the qualitative component by incorporating thematic summaries and illustrative quotes that better convey participant experiences and contextual factors related to malnutrition and food insecurity. (sections 2.5 paragraph 3, 3.1.4 and 3.1.5).

**Response to Reviewer #1**

**Comment 1:**

Correct the use of CD4 and be consistent with ART terminology.

**Response:**
Corrected as suggested. All instances of “CD4” have been standardized, and “HAART” has been replaced with “ART” throughout the manuscript.

**Comment 2:**

For Logistic regression, were interaction effects (e.g., gender × age) explored? Were multicollinearity and VIF checked?

**Response:**
Yes. We have now clarified in the **Data Analysis** section that we examined multicollinearity using the Variance Inflation Factor (VIF) and tolerance values, all within acceptable limits (<2.5). We also explored potential interaction effects (gender × age) and found no significant interactions. This information has been added to **Section 2.5**.

**Comment 3:**

Alcohol use seems unexpectedly high. Was there exploration of reasons for drinking and its effect on adherence or appointments?

**Response:**
We appreciate this important point. Qualitative responses indicated that occasional alcohol consumption was linked to peer influence and social gatherings. No significant relationship was found between alcohol use and ART adherence (**Section 3.1.4)**.

**Comment 4:**

Explore linkage between food preservation practices, water/sanitation variables, and malnutrition.

**Response:**
We have now included additional bivariate analyses assessing associations between food preservation, water source, and malnutrition indicators. No statistically significant associations were observed, but we have reported this finding briefly in **Section 3.2.4** and discussed the implications in **Section 4**.

**Comment 5:**

Consider caregiver perspectives to understand household dynamics.

**Response:**
We thank the reviewer for this insightful comment. Caregiver perspectives were not collected in this study; therefore, we were unable to explore the household-level dynamics from their viewpoint. We have clearly acknowledged this as a limitation in Section 4.1 (Strengths and Limitations), stating in the second paragraph: “excluding caregiver perspectives may have limited understanding of the broader household and social dynamics influencing adolescent nutrition. Caregivers play a pivotal role in food provision, treatment adherence, and emotional support, and their insights into food security and healthcare access could provide a more comprehensive view of factors shaping nutritional outcomes among ALHIV. Future studies should therefore include caregiver input to strengthen contextual understanding.”

**Response to Reviewer #2**

**Comment 1:**

Abstract: Replace “influencing” with “associated with.”

**Response:**
Corrected as suggested. The Abstract now states:
“This study identified factors associated with nutritional status among adolescents on ART…”

**Comment 2:**

Introduction: Support the statement “adolescents disproportionately bear the burden” with data or citation.

**Response:**
A supporting citation from UNAIDS (2023) has been added in the Introduction to substantiate this statement (section 2).

**Comment 3:**

Clarify if participants were hospitalized, outpatient, or both.

**Response:**
Clarified. All participants were **outpatients** attending ART clinics for routine follow-up (section 2.1).

**Comment 4:**

The meaning of “survey design” is unclear.

**Response:**
The explanation of study design was expanded to “ An institutional-based mixed-methods design was employed. The predominant quantitative component used a cross-sectional survey design, supplemented by clinical assessments and extraction of clinical record data. The survey consisted of a composite structured, interviewer-administered questionnaire.” (Section 2.2)

**Comment 5:**

Streamline overly detailed sections on sample size and data collection.

**Response:**
We have summarized these sections, retaining only key methodological information and moving detailed operational procedures to supplementary materials (section 2.3).

**Comment 6:**

Justify converting qualitative data into quantitative variables.

**Response:**
We added an explanation in Section 2.5 stating that qualitative responses on barriers (e.g., food insecurity, stigma, access to supplementation) were dichotomized to enable quantitative modeling. Additionally, thematic coding and direct quotes were later included to complement quantitative findings (sections 2.5 paragraph 3, 3.1.4 and 3.1.5).

**Comment 7:**

Clarify unclear sentence on ART initiation age.

**Response:**
 We revised the Results section for clarity, especially the statement on **age at ART initiation**, which now reads: “The median age at HIV diagnosis and ART initiation was 3.95 years (mean 4.66 ± 3.7 years). Half of the participants (50.5%, n=194) began ART before the age of 4” (section 3.1.3).

**Comment 8:**

Replace “determinant factors” with “associated factors” and avoid implying causality.

**Response:**
Implemented throughout. We also revised the Discussion to reflect that associations observed are non-causal and may be bidirectional.

**Comment 9:**

Clarify anemia–malnutrition directionality and avoid overstatement.

**Response:**
We have revised this as suggested. The text in section 4 now emphasizes that anemia may result from or coexist with malnutrition due to shared underlying factors (micronutrient deficiencies, infections, or ART side effects), and reads as: “Similarly, low haemoglobin levels were associated with malnutrition, consistent with evidence that anaemia and undernutrition frequently co-occur in HIV-infected population [50]. However, this cross-sectional design precludes establishing directionality; malnutrition may contribute to anemia through micronutrient deficiency, while anemia may also arise from infection or ART side effects”.

**Comment 10:**

Thematic presentation of qualitative findings could strengthen the paper.

**Response:**
We have developed our qualitative analysis thematically, added supporting quotes, and used them to reinforce key interpretations in the Discussion (sections 2.5 paragraph 3, 3.1.4, 3.1.5 and 4).

**Additional Revisions**

- All language and grammatical inconsistencies were corrected for clarity and conciseness.
- Figures were reformatted and validated through the PLOS NAAS tool as per journal requirements.
- The Inclusivity in Global Research questionnaire has been completed and uploaded as Supporting Information.

We hope that these extensive revisions address all concerns raised by the reviewers and the editor. We appreciate the opportunity to revise and resubmit our manuscript for further consideration in *PLOS Global Public Health*.

With kind regards,
**Dr. Meless Gebrie Bore**
(on behalf of all co-authors)
