# Supplementary material for: Clinical, lifestyle, environmental and dietary determinants of malnutrition in adolescents on antiretroviral therapy in Ethiopia
Source: PLOS Glob Public Health. 2026 Jun 26;6(6):e0005003. doi: 10.1371/journal.pgph.0005003 (PMC13309033; doi:10.1371/journal.pgph.0005003)
Supplement: Attachment — Submitted filename: Response to Reviewers.docx [file pgph.0005003.s011.docx]

**Response to Reviewers – PGPH-D-25-01939R1**

**Manuscript:** *Clinical, Lifestyle, Environmental and Dietary Determinants of Malnutrition in Adolescents on Antiretroviral Therapy in Ethiopia*
**Corresponding Author:** Meless Gebrie Bore

| **Comment (Reviewer/Editor)** | **Author Response** | **Action Taken in Manuscript** |
| --- | --- | --- |
| **Reviewer 1:** In Section 2.3 (Selection Criteria and Sampling Process), the exclusion criteria should explicitly include “inpatients.” | Thank you. We clarified that inpatients were excluded from the study. | Revised Section 2.3 to explicitly state: “Adolescents receiving inpatient care were excluded” (page. 5). |
| Correct all instances of “CD4” to uppercase. | Corrected all instances. | All “cd4” references changed to “CD4” throughout the manuscript. |
| Section 3.1.3: rephrase “… and only 1% of the participants had 150 copies/ml” to “… and only 1% of the participants had 150 copies/ml or more.” | Rephrased as suggested. | Updated Section 3.1.3 for clarity (page.11). |
| Paragraph beginning “Although quantitative analysis did not show significant associations…” is interpretive; better in Discussion. | Moved the paragraph to the Discussion section. | Section relocated to Discussion to avoid interpretation in Results (page. 18-22). |
| **Reviewer 3 / Editor:** Introduction: revise “This study aimed to examine clinical, lifestyle, environmental and dietary factors associated with the nutritional status of adolescents on ART in selected Ethiopian hospitals” to “…recruited as outpatients from selected Ethiopian hospitals.” | Revised to accurately reflect study population. | Updated Introduction sentence (page. 4). |
| Methods: Change inclusion/exclusion criteria to full sentences for clarity. | Revised for readability. | Rewrote inclusion/exclusion criteria as full sentences in Methods (page. 6). |
| Include validated measures for household food security and dietary diversity if used. | Clarified scales used: Household Food Insecurity Access Scale (HFIAS) and FAO dietary diversity score. | Added descriptions of validated tools in Methods (page. 8). |
| Avoid “increased with age” or “increased odds”; clarify cross-sectional associations. | Revised phrasing to reflect statistical associations rather than causal implications. | Updated all relevant sentences in Results to “was associated with” format ([page. 12]). |
| Define AOR and CI at first use. | Defined abbreviations in Results section. | Added “Adjusted Odds Ratio (AOR)” and “Confidence Interval (CI)” at first occurrence (page. 15). |
| Change interpretations of odds ratios from “more/less likely” to “higher/lower odds.” | Rephrased all OR interpretations according to reviewer guidance. | Updated Results section with recommended language (page. 15-19). |
| Section 3.2.1: “Food security was positively associated with thinness” → should be “Food insecurity” | Corrected text. | Revised Section 3.2.1 to indicate “Food insecurity was associated with higher odds of thinness” (page. 16). |
| Section 3.2.2: Sentence on cross-sectional nature unnecessary. | Removed redundant sentence. | Deleted sentence in Results (page. 18,). |
| Discussion: clarify if cited studies refer to ALHIV specifically. | Added clarification on whether comparative studies focused on ALHIV or adolescents more broadly. | Discussion revised with explicit population context (page. 19). |
| Consider discussing mental health, malnutrition, and stigma in relation to quantitative and qualitative findings. | Added discussion linking malnutrition with anxiety, internalized stigma, and mental health, and implications for school-based nutrition interventions. | Expanded Discussion to integrate mental health and stigma considerations (page. 22). |
| Copyedit for punctuation and minor errors. | Manuscript thoroughly copyedited. | Minor grammatical, punctuation, and formatting corrections applied throughout manuscript |
