# Supplementary material for: Clinical, lifestyle, environmental and dietary determinants of malnutrition in adolescents on antiretroviral therapy in Ethiopia
Source: PLOS Glob Public Health. 2026 Jun 26;6(6):e0005003. doi: 10.1371/journal.pgph.0005003 (PMC13309033; doi:10.1371/journal.pgph.0005003)
Supplement: Attachment — Submitted filename: Response to Editors V4.docx [file pgph.0005003.s012.docx]

**Response to Editorial Office Comments**

Manuscript ID: PGPH-D-25-01939R3
Title: Clinical, Lifestyle, Environmental and Dietary Determinants of Malnutrition in Adolescents on Antiretroviral Therapy in Ethiopia

Dear Mr. Tolentino,

Thank you for your email and for the opportunity to revise our manuscript. We have carefully addressed all the issues raised and revised the manuscript accordingly. Our detailed responses are provided below:

1. Financial Disclosure Statement
   We have provided a complete and detailed Financial Disclosure statement in full sentences, including the source of funding, the recipient (MGB), and the role of the funder. The statement has been updated in both the manuscript and the submission system to ensure consistency.
2. Consistency of Funding Information
   We have verified that the funding information, including the funder name and details, is consistent between the Financial Disclosure section and the Funding Information tab in the submission system.
3. Data Availability Statement
   We have revised the Data Availability statement to clearly explain the ethical restrictions on data sharing due to sensitive human participant information. The statement now specifies the responsible ethics committees and provides contact details for data access requests.
4. Figures
   All main figures have been provided as separate files in .tif format, with appropriate resolution and file size in accordance with the journal’s guidelines.
5. Supporting Information Legends
   A complete list of Supporting Information legends (S1–S6 Tables) has been added to the manuscript, and all corresponding files have been uploaded as separate Supporting Information files.

We have rebuilt and reviewed the PDF and confirm that all required sections are correctly included. We have approved the submission in the system.

We appreciate your consideration and look forward to the next stage of the review process.

Kind regards,
Dr. Meless Gebrie Bore
(on behalf of all authors)
