# Supplementary material for: Clinical, lifestyle, environmental and dietary determinants of malnutrition in adolescents on antiretroviral therapy in Ethiopia
Source: PLOS Glob Public Health. 2026 Jun 26;6(6):e0005003. doi: 10.1371/journal.pgph.0005003 (PMC13309033; doi:10.1371/journal.pgph.0005003)
Supplement: Attachment — Submitted filename: Response to reviewer or Editor.docx [file pgph.0005003.s013.docx]

**Reviewer/Editor Response**

| **Reviewer/Editor Comment** | **Concise Response** | **Location in Revised Manuscript** |
| --- | --- | --- |
| Harmonise font type throughout the manuscript | The font type and formatting were harmonised throughout the manuscript, tables, figures, and supplementary materials. | Throughout manuscript |
| Abstract: results section: please include odds ratios for factors associated | Odds ratios and 95% confidence intervals for significant factors associated with malnutrition were added to the Abstract results section. | Abstract, page 2-3 |
| Ethics approval: Please have this placed in the right position of the manuscript | The Ethics approval section was repositioned to the end of the Methods section in accordance with journal structure requirements. | Section 2.6, page 12-13 |
| Introduction: Please provide a reference for the sentence, “Globally, HIV/AIDS remains a significant public health challenge,…” | Appropriate references were added to support the statement. | Introduction, page 4-5 |
| State the actual statistics of the proportions of PLHIV made up by adolescents | The proportion of adolescents among people living with HIV was added to the Introduction with supporting references. | Introduction, page 4-5 |
| State the definition of adolescence age group and state reference | The WHO definition of adolescence (10–19 years) and corresponding reference were added. | Introduction, page 4 |
| What is the burden of malnutrition among adolescents in Ethiopia? | Additional epidemiological evidence on the burden of adolescent malnutrition in Ethiopia was added to the Introduction. | Introduction, page 4-5 |
| What proportion of adolescents on ART are experiencing comorbidities/virological non-suppression and stigma? | Relevant evidence regarding comorbidities, virological non-suppression, and stigma among adolescents on ART was added to the Introduction. | Introduction, page 4-5 |
| Provide more detail on the selected public health facilities such as location (rural vs urban vs peri-urban), HIV prevalence rates, HIV transmission rates and total clinic enrolments. | Additional information regarding hospital locations, HIV burden context, HIV transmission patterns, and total ART enrolment was added to the Study setting section. | Section 2.1, page 6 |
| What approach was used to triangulate quantitative and qualitative data? | The manuscript now explicitly states that a mixed-methods sequential explanatory design was used, with integration occurring during interpretation of findings. | Section 2.2, page 6 |
| Were the study tools adapted from previously published tools or developed specifically for the study? | We clarified that the questionnaire was adapted from previously validated instruments and published literature, and references were added accordingly. | Section 2.4.1, page 9 |
| What was the rationale for excluding adolescents on ART for less than 3 months? | The rationale for excluding adolescents on ART for less than three months was clarified in the Methods section. | Section 2.3, page 7 - 8 |
| Please illustrate the sample size calculation. | The sample size calculation formula, assumptions, and final sample size determination were added. | Section 2.3 and Supplementary File Box 2 |
| Provide a sample size allocation table to demonstrate the proportionate allocation of the sample size. | Probability proportional to size (PPS) allocation details and the sample allocation table were added in Supplementary File Box 2. | Supplementary File Box 2 |
| Under section 2.4 Data collection, the content of the first paragraph is best presented under Ethical consideration. | Ethical and consent-related information was moved to the Ethics section, and Section 2.4 was revised accordingly. | Sections 2.4 and 2.6, page 8 |
| Please clarify whether the healthcare professionals who collected the study data were not involved in routine service provision to the respondents. | We clarified that data collectors were not involved in the routine clinical care of participants. | Section 2.4, page 8 |
| Please clarify whether the respondents provided informed consent prior to data collection. Were there instances where assent and/or parental consent were required? | The manuscript now clarifies that written informed consent, parental/guardian consent, and adolescent assent were obtained prior to data collection where applicable. | Section 2.6, page 12 - 13 |
| State the brands of weighing scales that were used in the study | The brand and manufacturer of the weighing scales were already stated and retained in the revised manuscript. | Section 2.4.1, page 9 |
| Section 2.4.1: please provide references of the literature from which the data tools were adapted. | References for validated assessment tools and adapted instruments were added to Section 2.4.1. | Section 2.4.1, page 9 - 10 |
| Provide definitions of the signs of malnutrition that were assessed for from physical assessments | Operational definitions of the assessed signs of malnutrition were added according to WHO nutritional assessment guidelines. | Section 2.4.1, page 10 |
| Are viral load assessments part of the baseline assessments at ART initiation? | We clarified that clinical records included baseline and follow-up HIV monitoring indicators, including CD4 counts and viral load measurements. | Section 2.4.1, page 10 |
| Under section 2.4.1: Check the second last paragraph and remove repetition of “was assessed” | The repetitive wording was revised for clarity and readability. | Section 2.4.1, page 9 - 10 |
| How is it possible that the bivariate analysis utilized binary logistic regression yet the outcome was not a binary variable? | We clarified that binary outcome variables were created for regression analyses, making binary logistic regression appropriate. | Section 2.5, page 11 - 12 |
| Were the respondents for the qualitative and quantitative interviews similar? | We clarified that the same participants contributed to both the quantitative and qualitative components. | Section 2.4, page 8 |
| What was the theoretical basis for the themes that guided qualitative analysis? | We clarified that reflexive thematic analysis informed by Braun and Clarke’s approach guided qualitative analysis. | Section 2.5, page 12 |
| Did the team use any software for qualitative analysis? | We clarified that qualitative responses were analysed manually without specialised qualitative analysis software. | Section 2.5, page 12 |
| Section 3.1.3 Clinical Characteristics (HIV-related information): Please re-write, “Half of the participants……” to “More than half of the participants….. before the age of four years.” | The sentence was revised accordingly. | Section 3.1.3, page 14 |
| In paragraph 2, please correct, “at least one other HIV-positive family members..” to “at least one family member who was HIV positive” | The sentence was corrected as suggested. | Section 3.1.3, page 14 |
| Viral load results are presented for 98.7% of participants, what about the 1.3%? | We clarified that viral load data were unavailable for 1.3% of participants because laboratory results were not documented in clinical records at the time of data extraction. | Section 3.1.3, page 15 |
| Please remove bullets in paragraphs in section on mental health | The mental health section was revised into narrative paragraph format. | Section 3.1.4, page 15 |
| Reviewer 3: Need to add “of” in the first sentence after “experiences” | The sentence was corrected accordingly. | Section 3.2.3, page 21 |
| In the discussion section, first paragraph, include a sentence or two on the factors associated with malnutrition. | A summary of the significant factors associated with malnutrition was added to the first paragraph of the Discussion section. | Discussion, page 22 |
| Reviewer 3: In the Discussion paragraph beginning “Gender-based differences”, change interpretation of odds ratio to “male participants had significantly higher odds of being thin and acutely malnourished” | The interpretation of odds ratios was revised as suggested. | Discussion, page 23 |
