# Supplementary material for: Clinical, lifestyle, environmental and dietary determinants of malnutrition in adolescents on antiretroviral therapy in Ethiopia
Source: PLOS Glob Public Health. 2026 Jun 26;6(6):e0005003. doi: 10.1371/journal.pgph.0005003 (PMC13309033; doi:10.1371/journal.pgph.0005003)
Supplement: Peer review history

# Peer review: Decision Letter 0

**DOI:** 10.1371/journal.pgph.0005003.r001

**Authors:** Lina Taing

3 Oct 2025

PGPH-D-25-01939

Clinical, Lifestyle, Environmental and Dietary Determinants of Malnutrition in Adolescents on Antiretroviral Therapy in Ethiopia

PLOS Global Public Health

Dear Dr. Bore,

Thank you for submitting your manuscript to PLOS Global Public Health. After careful consideration, we feel that it has merit but does not fully meet PLOS Global Public Health’s publication criteria as it currently stands. Therefore, we invite you to submit a revised version of the manuscript that addresses the points raised during the review process.

Please submit your revised manuscript by Nov 17 2025 11:59PM. If you will need more time than this to complete your revisions, please reply to this message or contact the journal office at globalpubhealth@plos.org. When you're ready to submit your revision, log on to https://www.editorialmanager.com/pgph/ and select the 'Submissions Needing Revision' folder to locate your manuscript file.

Please include the following items when submitting your revised manuscript:

- A rebuttal letter that responds to each point raised by the editor and reviewer(s). You should upload this letter as a separate file labeled 'Response to Reviewers'.
- A marked-up copy of your manuscript that highlights changes made to the original version. You should upload this as a separate file labeled 'Revised Manuscript with Track Changes'.
- An unmarked version of your revised paper without tracked changes. You should upload this as a separate file labeled 'Manuscript'.

Guidelines for resubmitting your figure files are available below the reviewer comments at the end of this letter.

We look forward to receiving your revised manuscript.

Kind regards,

Lina Taing

Academic Editor

PLOS Global Public Health

Journal Requirements:

1. Please include a complete copy of PLOS’ questionnaire on inclusivity in global research in your revised manuscript. Our policy for research in this area aims to improve transparency in the reporting of research performed outside of researchers’ own country or community. The policy applies to researchers who have travelled to a different country to conduct research, research with Indigenous populations or their lands, and research on cultural artefacts. The questionnaire can also be requested at the journal’s discretion for any other submissions, even if these conditions are not met.  Please find more information on the policy and a link to download a blank copy of the questionnaire here: https://journals.plos.org/globalpublichealth/s/best-practices-in-research-reporting. Please upload a completed version of your questionnaire as Supporting Information when you resubmit your manuscript.

2. We ask that a manuscript source file is provided at Revision. Please upload your manuscript file as a .doc, .docx, .rtf or .tex.

3. Please provide a/amend your detailed Financial Disclosure statement. This is published with the article. It must therefore be completed in full sentences and contain the exact wording you wish to be published.

**Please only choose the relevant sentences from below**

a. Please clarify all sources of funding (financial or material support) for your study. List the grants (with grant number) or organizations (with url) that supported your study, including funding received from your institution.

b. State the initials, alongside each funding source, of each author to receive each grant.

c. State what role the funders took in the study. If the funders had no role in your study, please state: “The funders had no role in study design, data collection and analysis, decision to publish, or preparation of the manuscript.”

d. If any authors received a salary from any of your funders, please state which authors and which funders.

If you did not receive any funding for this study, please simply state: “The authors received no specific funding for this work.”

4. In the online submission form, you indicated that “The data supporting the findings of this study are available upon reasonable request. Access to the data may be restricted to ensure participant confidentiality and to comply with ethical guidelines.”.

All PLOS journals now require all data underlying the findings described in their manuscript to be freely available to other researchers, either

a. In a public repository,

b. Within the manuscript itself, or

c. Uploaded as supplementary information.

This policy applies to all data except where public deposition would breach compliance with the protocol approved by your research ethics board. If your data cannot be made publicly available for ethical or legal reasons (e.g., public availability would compromise patient privacy), please explain your reasons by return email and your exemption request will be escalated to the editor for approval. Your exemption request will be handled independently and will not hold up the peer review process, but will need to be resolved should your manuscript be accepted for publication. One of the Editorial team will then be in touch if there are any issues.

5. If the reviewer comments include a recommendation to cite specific previously published works, please review and evaluate these publications to determine whether they are relevant and should be cited. There is no requirement to cite these works unless the editor has indicated otherwise.

Additional Editor Comments (if provided):

This mixed-methods study of 384 Ethiopian adolescents living with HIV on antiretroviral therapy reveals high rates of malnutrition (24.2% thinness, 21.7% stunting, 34.9% acute malnutrition) and identifies key determinants including gender, age, chronic infections, mental health status, food insecurity, and nutritional supplementation. Strengths include your methodology (validated instruments, STROBE-Nut adherence, appropriate sample size calculations), assessment across multiple domains, and strong practical relevance—most participants were students, highlighting opportunities for school-based interventions.

However, the study has important limitations

- Methods: It is unclear whether participants were recruited from inpatient or outpatient settings; the description of “survey design” lacks precision; details on sample size and data collection are overly long and could be streamlined; and the decision to recode qualitative data into quantitative variables is unconventional and should be justified.

- Results: Some sentences (e.g., age at ART initiation) are unclear; the repeated use of “determinant factors” implies causality, which the cross-sectional design does not support; and certain associations (e.g., between TB and malnutrition, or anxiety and thinness) may be bidirectional or confounded.

- Discussion: Interpretations sometimes overstate directionality (e.g., malnutrition and anemia) or present unsurprising correlations (e.g., food insecurity and malnutrition) as novel; and translating qualitative responses into quantitative variables limited the richness of insights—more thematic coding and illustrative quotes could have strengthened the findings.

Recommendation: The authors should clarify methodological descriptions, carefully temper causal language, revise ambiguous reporting, and better integrate qualitative findings to deepen interpretation.

[Note: HTML markup is below. Please do not edit.]

Reviewers' comments:

Reviewer's Responses to Questions

**Comments to the Author**

1. Does this manuscript meet PLOS Global Public Health’s publication criteria? Is the manuscript technically sound, and do the data support the conclusions? The manuscript must describe methodologically and ethically rigorous research with conclusions that are appropriately drawn based on the data presented.

Reviewer #1: Yes

Reviewer #2: No

**********

2. Has the statistical analysis been performed appropriately and rigorously?

Reviewer #1: Yes

Reviewer #2: I don't know

**********

3. Have the authors made all data underlying the findings in their manuscript fully available (please refer to the Data Availability Statement at the start of the manuscript PDF file)?

The PLOS Data policy requires authors to make all data underlying the findings described in their manuscript fully available without restriction, with rare exception. The data should be provided as part of the manuscript or its supporting information, or deposited to a public repository. For example, in addition to summary statistics, the data points behind means, medians and variance measures should be available. If there are restrictions on publicly sharing data—e.g. participant privacy or use of data from a third party—those must be specified.

Reviewer #1: Yes

Reviewer #2: No

**********

4. Is the manuscript presented in an intelligible fashion and written in standard English?

PLOS Global Public Health does not copyedit accepted manuscripts, so the language in submitted articles must be clear, correct, and unambiguous. Any typographical or grammatical errors should be corrected at revision, so please note any specific errors here.

Reviewer #1: Yes

Reviewer #2: Yes

**********

5. Review Comments to the Author

Please use the space provided to explain your answers to the questions above. You may also include additional comments for the author, including concerns about dual publication, research ethics, or publication ethics. (Please upload your review as an attachment if it exceeds 20,000 characters)

Reviewer #1: 1. Congratulations to the researchers for their work on malnutrition in adolescents living with HIV on treatment. The study is clear and very structured, statistically powered for generalizability and uses validated standard anthropometric tools. The study identifies multifaceted determinants after controlling for confounders. Most importantly, It underscores actionable insights, such as the role of mental health and food insecurity, which could inform policy in low-resource settings like Ethiopia.

2. Under 2.4.1 Components of the questionnaire: The sentence with reference [21] on page 11 of 43 should have CD4 corrected accordingly. Similarly correct same in page 14 of 43 under HIV-related information and please be consistent with the use of ART and HAART. Note HAART is no longer commonly used and so ART is more preferred.

3. Under 2.5 Data processing and analysis: For Logistic regression analysis, I wanted to find out if for model robustness, interaction effects for instance gender × age were explored? And similarly, checks for multicollinearity and calculating for Variance Inflation Factor (VIF)?

4. Under 3.1.4 Lifestyle-related characteristics - alcohol use: The level of alcohol usage was a bit quite surprising to me. Was there an exploration of why they were drinking and whether that impacted their adherence in any way or missing appointments?

5. It would be good to explore the linkage of the data collected on variables considered for food preservation practices to the nutritional outcomes of interest for the study participants. Similarly explore if there was any statistical association on the variables for water and sanitation with malnutritional outcomes.

6. A good discussion on family size and household. I wanted to find out the extent to which care giver perspectives explored to understand household dynamics.

Reviewer #2: Thank you to the authors for this submission and for drawing attention to the important topic of malnutrition in ALHIV in Ethiopia. The findings of this study as it is currently framed seem to have limited novel contribution to the scientific literature. More insightful findings may be gained by focusing more on the high prevalence of malnutrition in this population and by analysing the qualitative data in through thematic coding with the presentation of illustrative quotes, to better demonstrate the nature of the issue in this population and identify potential solutions in this setting.

Abstract :

1. Background : Authors mention factors « influencing » nutritional status but the study design supports finding factors associated with nutritional status at best—the study design does not support causal findings or even suggesting a direction of association.

Introduction :

1. Authors state « adolescents disproportionately bear the burden of the disease. » Please consider including a figure or citation to support this assertion.

Methods :

1. Authors mention patients being recruited from hospitalized. Please consider specifying if patients are currently hospitalized, being followed outpatient, or a mix of both.

2. In 2.2 the quthors mention : « The quantitative component primarily utilized survey design, supplemented by clinical assessments and the extraction of data from health records. ». This description of the quantitative methods is insufficient. The meaning of « survey design » as a method is unclear.

3. In 2.4 the authors provide more detail than necessary on the sample size calculation and data collection methods. This can be summarized and shortened.

4. In 2.5 Authors state : « Qualitative data were coded into categorical variables and integrated with the quantitative dataset. » This is generally not a best practice with qualitative data. Authors should explain why this was chosen over a more traditional approach to exploring qualitative data (inductive or deductive coding for themes, etc).

Results :

1. In 3.13 the authors mention : « Of the 384 ALHIV participating in the study, 50.5% (n=194) had been diagnosed and commenced ART at the (median) age of 3.95 years/mean (SD) age 4.66 ± 3.7 years. » This sentence is not clear. Half of the partiipants had been diagnosed all at the media nage of 3.95 years ? Please consider revising.

2. In 3.2 and other sections, the authors refer to « determinant factors for thinness ». The study design allows for identification of correlation, whereas the term « determinant » implies causation. The authors should consider taking extreme care to avoid overstating any potential causal relationship or direction of causality. For example, while TB and malnutrition are associated, the relationsihp is widely considered to be bidirectional. Likewise, what within the study allows for the conclusion that anxiety is leading to thinness, and not the reverse, or that anxiety may be related with another variable in the model such as lack of access to food or low income ?

Discussion :

1. The authors state « Additionally, haemoglobin levels were found to be a strong predictor of malnutrition, with lower levels significantly increasing the likelihood of nutritional deficiencies. » However, the causal arrow between malnutrition and anemia is generally considered to go in the other direction—as mentioned in reference 47 stated by the authors, « HIV-related anemia may be as a result of opportunistic infections, side effect of chemotherapeutic treatment, changes in cytokine expression with a resultant decrease in blood cell production, and a consequence of micronutrient deficiencies ». The authors should take care in interpreting the directionality and relevance of any associations identified.

2. Overall the findings on correlation between certain factors and malnutrition do not appear to be very unexpected or insightful (such as correlation between lack of food and malnutrition). Other times the causal relationship is overstated, despite authors acknowledging in the limitations that the study design doese not support conclusions around causation.

3. Translating the qualitative responses into quantitative variables was a missed opportunity. I wonder if the qualitative responses could have better supported the suggested ways forward proposed by the authors if they had been coded for themes and presented as quotes.

**********

6. PLOS authors have the option to publish the peer review history of their article (what does this mean?). If published, this will include your full peer review and any attached files.

**Do you want your identity to be public for this peer review?**  If you choose “no”, your identity will remain anonymous but your review may still be made public.

For information about this choice, including consent withdrawal, please see our Privacy Policy.

Reviewer #1: No

Reviewer #2: No

**********

[NOTE: If reviewer comments were submitted as an attachment file, they will be attached to this email and accessible via the submission site. Please log into your account, locate the manuscript record, and check for the action link "View Attachments". If this link does not appear, there are no attachment files.]

-->Figure Resubmissions:

While revising your submission, we strongly recommend that you use PLOS’s NAAS tool (https://ngplosjournals.pagemajik.ai/artanalysis) to test your figure files. NAAS can convert your figure files to the TIFF file type and meet basic requirements (such as print size, resolution), or provide you with a report on issues that do not meet our requirements and that NAAS cannot fix.

After uploading your figures to PLOS’s NAAS tool - https://ngplosjournals.pagemajik.ai/artanalysis, NAAS will process the files provided and display the results in the "Uploaded Files" section of the page as the processing is complete. If the uploaded figures meet our requirements (or NAAS is able to fix the files to meet our requirements), the figure will be marked as "fixed" above. If NAAS is unable to fix the files, a red "failed" label will appear above. When NAAS has confirmed that the figure files meet our requirements, please download the file via the download option, and include these NAAS processed figure files when submitting your revised manuscript.

# Author comment: Author response to Decision Letter 1

**DOI:** 10.1371/journal.pgph.0005003.r002

1 Dec 2025

Attachment Submitted filename: Response to Editor and Reviewers questions.docx

# Peer review: Decision Letter 1

**DOI:** 10.1371/journal.pgph.0005003.r003

**Authors:** Lina Taing, Helen Howard

13 Jan 2026

PGPH-D-25-01939R1

Clinical, Lifestyle, Environmental and Dietary Determinants of Malnutrition in Adolescents on Antiretroviral Therapy in Ethiopia

PLOS Global Public Health

Dear Dr. Bore,

Thank you for submitting your manuscript to PLOS Global Public Health. After careful consideration, we feel that it has merit but does not fully meet PLOS Global Public Health’s publication criteria as it currently stands. Therefore, we invite you to submit a revised version of the manuscript that addresses the points raised during the review process.

Please submit your revised manuscript by Feb 27 2026 11:59PM. If you will need more time than this to complete your revisions, please reply to this message or contact the journal office at globalpubhealth@plos.org. When you're ready to submit your revision, log on to https://www.editorialmanager.com/pgph/ and select the 'Submissions Needing Revision' folder to locate your manuscript file.

Please include the following items when submitting your revised manuscript:

- A letter that responds to each point raised by the editor and reviewer(s). You should upload this letter as a separate file labeled 'Response to Reviewers'.
- A marked-up copy of your manuscript that highlights changes made to the original version. You should upload this as a separate file labeled 'Revised Manuscript with Track Changes'.
- An unmarked version of your revised paper without tracked changes. You should upload this as a separate file labeled 'Manuscript'.

Guidelines for resubmitting your figure files are available below the reviewer comments at the end of this letter.

We look forward to receiving your revised manuscript.

Kind regards,

Helen Howard

Staff Editor

PLOS Global Public Health

Journal Requirements:

1. Please include a complete copy of PLOS’ questionnaire on inclusivity in global research in your revised manuscript. Our policy for research in this area aims to improve transparency in the reporting of research performed outside of researchers’ own country or community. The policy applies to researchers who have travelled to a different country to conduct research, research with Indigenous populations or their lands, and research on cultural artefacts. The questionnaire can also be requested at the journal’s discretion for any other submissions, even if these conditions are not met.  Please find more information on the policy and a link to download a blank copy of the questionnaire here: https://journals.plos.org/globalpublichealth/s/best-practices-in-research-reporting. Please upload a completed version of your questionnaire as Supporting Information when you resubmit your manuscript.

If the reviewer comments include a recommendation to cite specific previously published works, please review and evaluate these publications to determine whether they are relevant and should be cited. There is no requirement to cite these works unless the editor has indicated otherwise.

Additional Editor Comments (if provided):

[Note: HTML markup is below. Please do not edit.]

Reviewers' comments:

Reviewer's Responses to Questions

**Comments to the Author**

1. If the authors have adequately addressed your comments raised in a previous round of review and you feel that this manuscript is now acceptable for publication, you may indicate that here to bypass the “Comments to the Author” section, enter your conflict of interest statement in the “Confidential to Editor” section, and submit your "Accept" recommendation.

Reviewer #1: All comments have been addressed

Reviewer #3: (No Response)

**********

2. Does this manuscript meet PLOS Global Public Health’s publication criteria? Is the manuscript technically sound, and do the data support the conclusions? The manuscript must describe methodologically and ethically rigorous research with conclusions that are appropriately drawn based on the data presented.

Reviewer #1: Yes

Reviewer #3: Partly

**********

3. Has the statistical analysis been performed appropriately and rigorously?

Reviewer #1: Yes

Reviewer #3: Yes

**********

4. Have the authors made all data underlying the findings in their manuscript fully available (please refer to the Data Availability Statement at the start of the manuscript PDF file)?

The PLOS Data policy requires authors to make all data underlying the findings described in their manuscript fully available without restriction, with rare exception. The data should be provided as part of the manuscript or its supporting information, or deposited to a public repository. For example, in addition to summary statistics, the data points behind means, medians and variance measures should be available. If there are restrictions on publicly sharing data—e.g. participant privacy or use of data from a third party—those must be specified.

Reviewer #1: Yes

Reviewer #3: No

**********

5. Is the manuscript presented in an intelligible fashion and written in standard English?

PLOS Global Public Health does not copyedit accepted manuscripts, so the language in submitted articles must be clear, correct, and unambiguous. Any typographical or grammatical errors should be corrected at revision, so please note any specific errors here.

Reviewer #1: Yes

Reviewer #3: Yes

**********

6. Review Comments to the Author

Please use the space provided to explain your answers to the questions above. You may also include additional comments for the author, including concerns about dual publication, research ethics, or publication ethics. (Please upload your review as an attachment if it exceeds 20,000 characters)

Reviewer #1: All previous comments have been fully addressed, and the manuscript has been substantially revised for clarity and conciseness.

Methodology

i)In Section 2.3 (Selection Criteria and Sampling Process), the exclusion criteria should explicitly include “inpatients,” given that the study setting and period clearly state that no inpatients were included.

ii)Please correct all instances to “CD4” (uppercase).

Results

i)Page 15, Section 3.1.3: Consider rephrasing “… and only 1% of the participants had 150 copies/ml” to “… and only 1% of the participants had 150 copies/ml or more.”

ii)Please clarify why the paragraph beginning with “Although quantitative analysis did not show significant associations…” is presented under the Results section. The content reads as interpretation and may be more appropriately placed in the Discussion.

Reviewer #3: Comments from editor and reveiwers #1 and #2 were addressed well. I have some additional comments.

Introduction

"This study aimed to examine clinical, lifestyle, environmental and dietary factors associated with the

nutritional status of adolescents on ART in selected Ethiopian hospitals." In line with edits, change to “recruited as outpatients from selected Ethiopian hospitals.”

Methods:

Consider changing inclusion/exclusion to full sentences for the reader.

Was a validated measure of household food security used? If yes, include the name of the scale/score. Same for dietary diversity.

Results:

Avoid using the phrase “increased with age” or “increased odds” as this was not a longitudinal study. Something like “anxiety was associated with older age” or “Low haemoglobin levels (<11.5 g/dl) were associated with higher odds of malnutrition” would be more appropriate.

Words missing in first two sentences in 3.1.5, and the third sentence contains presumably extra words

Define AOR and CI at first uses.

For odds ratios, change all interpretations from “more/less likely” to “lower/higher odds.” Example: “Female adolescents living with HIV (ALHIV) had 73% lower odds of being thin compared

to their male counterparts (AOR = 0.27; 95% CI: 0.16, 0.45).” Another way to interpret, using a different example result, could be “ALHIV experiencing food insecurity had 1.63 times the odds of being thin compared to those without food insecurity (AOR = 1.63; 95%

CI: 1.02, 2.62).” or “Food insecurity was associated with 1.63 times the odds of being thin compared to being food secure (AOR = 1.63; 95% CI: 1.02, 2.62).

3.2.1. For this sentence: “Food security was positively associated with thinness.” I think you mean to say “Food insecurity” here.

3.2.2. I find the sentence “As the study was cross-sectional, these findings represent statistical association rather than causal relationship.” To be unnecessary, especially given you adequately noted this in the discussion section.

Discussion

Second paragraph: Are the studies you are describing of ALHIV? I would clarify. If adolescents more broadly, note that and explain why your study might show higher/lower stunting/thinness comparatively.

A thorough copyedit would help catch punctuation errors resulting from revision through tracked changes.

It may be interesting to discuss your results related to mental health, malnutrition, and stigma given the association of malnutrition and mental health problems from the quant data and that your qualitative findings illustrated shame/ internalized stigma related to food insecurity. This should also be taken into consideration for your proposed school-based nutrition intervention.

**********

7. PLOS authors have the option to publish the peer review history of their article (what does this mean?). If published, this will include your full peer review and any attached files.

**Do you want your identity to be public for this peer review?**  If you choose “no”, your identity will remain anonymous but your review may still be made public.

For information about this choice, including consent withdrawal, please see our Privacy Policy.

Reviewer #1: No

Reviewer #3: No

**********

[NOTE: If reviewer comments were submitted as an attachment file, they will be attached to this email and accessible via the submission site. Please log into your account, locate the manuscript record, and check for the action link "View Attachments". If this link does not appear, there are no attachment files.]

-->Figure Resubmissions:

While revising your submission, we strongly recommend that you use PLOS’s NAAS tool (https://ngplosjournals.pagemajik.ai/artanalysis) to test your figure files. NAAS can convert your figure files to the TIFF file type and meet basic requirements (such as print size, resolution), or provide you with a report on issues that do not meet our requirements and that NAAS cannot fix.

After uploading your figures to PLOS’s NAAS tool - https://ngplosjournals.pagemajik.ai/artanalysis, NAAS will process the files provided and display the results in the "Uploaded Files" section of the page as the processing is complete. If the uploaded figures meet our requirements (or NAAS is able to fix the files to meet our requirements), the figure will be marked as "fixed" above. If NAAS is unable to fix the files, a red "failed" label will appear above. When NAAS has confirmed that the figure files meet our requirements, please download the file via the download option, and include these NAAS processed figure files when submitting your revised manuscript.

# Author comment: Author response to Decision Letter 2

**DOI:** 10.1371/journal.pgph.0005003.r004

26 Jan 2026

Attachment Submitted filename: Response to Reviewers.docx

# Peer review: Decision Letter 2

**DOI:** 10.1371/journal.pgph.0005003.r005

**Authors:** Lina Taing, Helen Howard, Andrew Kazibwe

11 Feb 2026

PGPH-D-25-01939R2

Clinical, Lifestyle, Environmental and Dietary Determinants of Malnutrition in Adolescents on Antiretroviral Therapy in Ethiopia

PLOS Global Public Health

Dear Dr. Bore,

Thank you for submitting your manuscript to PLOS Global Public Health. After careful consideration, we feel that it has merit but does not fully meet PLOS Global Public Health’s publication criteria as it currently stands. Therefore, we invite you to submit a revised version of the manuscript that addresses the points raised during the review process.

==============================

While majority of the comments from the previous round of review have been satisfactorily addressed, there are a few additional observations that require your attention.

==============================

Please submit your revised manuscript by Mar 13 2026 11:59PM. If you will need more time than this to complete your revisions, please reply to this message or contact the journal office at globalpubhealth@plos.org. When you're ready to submit your revision, log on to https://www.editorialmanager.com/pgph/ and select the 'Submissions Needing Revision' folder to locate your manuscript file.

Please include the following items when submitting your revised manuscript:

- A letter that responds to each point raised by the editor and reviewer(s). You should upload this letter as a separate file labeled 'Response to Reviewers'.
- A marked-up copy of your manuscript that highlights changes made to the original version. You should upload this as a separate file labeled 'Revised Manuscript with Track Changes'.
- An unmarked version of your revised paper without tracked changes. You should upload this as a separate file labeled 'Manuscript'.

Guidelines for resubmitting your figure files are available below the reviewer comments at the end of this letter.

We look forward to receiving your revised manuscript.

Kind regards,

Andrew Kazibwe, MBChB, MMED

Academic Editor

PLOS Global Public Health

Journal Requirements:

If the reviewer comments include a recommendation to cite specific previously published works, please review and evaluate these publications to determine whether they are relevant and should be cited. There is no requirement to cite these works unless the editor has indicated otherwise.

Please review your reference list to ensure that it is complete and correct. If you have cited papers that have been retracted, please include the rationale for doing so in the manuscript text, or remove these references and replace them with relevant current references. Any changes to the reference list should be mentioned in the rebuttal letter that accompanies your revised manuscript. If you need to cite a retracted article, indicate the article’s retracted status in the References list and also include a citation and full reference for the retraction notice.

Additional Editor Comments (if provided):

Reviewer 3

3.2.3.

Need to add “of” in the first sentence after “experiences”

In the Discussion paragraph beginning “Gender-based differences”, change interpretation of odds ratio to “male participants had significantly higher odds of being thin and acutely malnourished”

Reviewer 4

Harmonise font type throughout the manuscript

Abstract: results section: please include odds ratios for factors associated

Ethics approval: Please have this placed in the right position of the manuscript

Introduction: Please provide a reference for the sentence, “Globally, HIV/AIDS remains a significant public health challenge,…”

State the actual statistics of the proportions of PLHIV made up by adolescents

State the definition of adolescence age group and state reference

What is the burden of malnutrition among adolescents in Ethiopia?

What proportion of adolescents on ART are experiencing comorbidities/virological non-suppression and stigma?

Provide more detail on the selected public health facilities such as location (rural vs urban vs peri-urban), HIV prevalence rates, HIV transmission rates and total clinic enrolments.

What approach was used to triangulate quantitative and qualitative data? Was it a sequential explanatory, sequential exploratory or parallel model?

Were the study tools adapted from previously published tools or developed specifically for the study? If adopted, please provide a reference. If developed specifically for the study, state how they were validated.

What was the rationale for excluding adolescents on ART for less than 3 months?

Please illustrate the sample size calculation.

Provide a sample size allocation table to demonstrate the proportionate allocation of the sample size.

Under section 2.4 Data collection, the content of the first paragraph is best presented under Ethical consideration.

Please clarify whether the healthcare professionals who collected the study data were not involved in routine service provision to the respondents.

Please clarify whether the respondents provided informed consent prior to data collection. Were there instances where assent and/or parental consent were required?

State the brands of weighing scales that were used in the study

Section 2.4.1: please provide references of the literature from which the data tools were adapted.

Provide definitions of the signs of malnutrition that were assessed for from physical assessments

Are viral load assessments part of the baseline assessments at ART initiation?

Under section 2.4.1: Check the second last paragraph and remove repetition of “was assessed”

How is it possible that the bivariate analysis utilized binary logistic regression yet the outcome was not a binary variable?

Were the respondents for the qualitative and quantitative interviews similar?

What was the theoretical basis for the themes that guided qualitative analysis?

Did the team use any software for qualitative analysis?

Section 3.1.3 Clinical Characteristics (HIV-related information): Please re-write, “Half of the participants……” to “More than half of the participants….. before the age of four years.”

In paragraph 2, please correct, “at least one other HIV-positive family members..” to “at least one family member who was HIV positive”

Viral load results are presented for 98.7% of participants, what about the 1.3%?

Please remove bullets in paragraphs in section on mental health

In the discussion section, first paragraph, include a sentence or two on the factors associated with malnutrition

[Note: HTML markup is below. Please do not edit.]

Reviewers' comments:

Reviewer's Responses to Questions

**Comments to the Author**

1. If the authors have adequately addressed your comments raised in a previous round of review and you feel that this manuscript is now acceptable for publication, you may indicate that here to bypass the “Comments to the Author” section, enter your conflict of interest statement in the “Confidential to Editor” section, and submit your "Accept" recommendation.

Reviewer #1: All comments have been addressed

Reviewer #3: All comments have been addressed

**********

2. Does this manuscript meet PLOS Global Public Health’s publication criteria? Is the manuscript technically sound, and do the data support the conclusions? The manuscript must describe methodologically and ethically rigorous research with conclusions that are appropriately drawn based on the data presented.

Reviewer #1: Yes

Reviewer #3: Yes

**********

3. Has the statistical analysis been performed appropriately and rigorously?

Reviewer #1: Yes

Reviewer #3: Yes

**********

4. Have the authors made all data underlying the findings in their manuscript fully available (please refer to the Data Availability Statement at the start of the manuscript PDF file)?

The PLOS Data policy requires authors to make all data underlying the findings described in their manuscript fully available without restriction, with rare exception. The data should be provided as part of the manuscript or its supporting information, or deposited to a public repository. For example, in addition to summary statistics, the data points behind means, medians and variance measures should be available. If there are restrictions on publicly sharing data—e.g. participant privacy or use of data from a third party—those must be specified.

Reviewer #1: Yes

Reviewer #3: (No Response)

**********

5. Is the manuscript presented in an intelligible fashion and written in standard English?

PLOS Global Public Health does not copyedit accepted manuscripts, so the language in submitted articles must be clear, correct, and unambiguous. Any typographical or grammatical errors should be corrected at revision, so please note any specific errors here.

Reviewer #1: Yes

Reviewer #3: Yes

**********

6. Review Comments to the Author

Please use the space provided to explain your answers to the questions above. You may also include additional comments for the author, including concerns about dual publication, research ethics, or publication ethics. (Please upload your review as an attachment if it exceeds 20,000 characters)

Reviewer #1: Congratulations to the research team/authors.

Reviewer #3: 3.2.3.

Need to add “of” in the first sentence after “experiences”

In the Discussion paragraph beginning “Gender-based differences”, change interpretation of odds ratio to “male participants had significantly higher odds of being thin and acutely malnourished”

**********

7. PLOS authors have the option to publish the peer review history of their article (what does this mean?). If published, this will include your full peer review and any attached files.

**Do you want your identity to be public for this peer review?**  If you choose “no”, your identity will remain anonymous but your review may still be made public.

For information about this choice, including consent withdrawal, please see our Privacy Policy.

Reviewer #1: No

Reviewer #3: No

**********

[NOTE: If reviewer comments were submitted as an attachment file, they will be attached to this email and accessible via the submission site. Please log into your account, locate the manuscript record, and check for the action link "View Attachments". If this link does not appear, there are no attachment files.]

Figure Resubmissions:

While revising your submission, we strongly recommend that you use PLOS’s NAAS tool (https://ngplosjournals.pagemajik.ai/artanalysis) to test your figure files. NAAS can convert your figure files to the TIFF file type and meet basic requirements (such as print size, resolution), or provide you with a report on issues that do not meet our requirements and that NAAS cannot fix.

After uploading your figures to PLOS’s NAAS tool - https://ngplosjournals.pagemajik.ai/artanalysis, NAAS will process the files provided and display the results in the "Uploaded Files" section of the page as the processing is complete. If the uploaded figures meet our requirements (or NAAS is able to fix the files to meet our requirements), the figure will be marked as "fixed" above. If NAAS is unable to fix the files, a red "failed" label will appear above. When NAAS has confirmed that the figure files meet our requirements, please download the file via the download option, and include these NAAS processed figure files when submitting your revised manuscript.

# Author comment: Author response to Decision Letter 3

**DOI:** 10.1371/journal.pgph.0005003.r006

20 Mar 2026

Attachment Submitted filename: Response to Editors V4.docx

# Editor report: Decision Letter 3

**DOI:** 10.1371/journal.pgph.0005003.r007

**Authors:** Lina Taing, Helen Howard, Andrew Kazibwe, Ilse Bloom

30 Apr 2026

PGPH-D-25-01939R3

Clinical, Lifestyle, Environmental and Dietary Determinants of Malnutrition in Adolescents on Antiretroviral Therapy in Ethiopia

PLOS Global Public Health

Dear Dr. Bore,

Thank you for submitting your manuscript to PLOS Global Public Health. After careful consideration, we feel that it has merit but does not fully meet PLOS Global Public Health’s publication criteria as it currently stands. Therefore, we invite you to submit a revised version of the manuscript that addresses the points raised during the review process.

Please see below the comments from the Academic Editor. These comments were included in the previous decision letter but they do not appear to have been addressed.

Could you please carefully revise the manuscript to address all comments raised?

**Academic Editor Comments:**

Harmonise font type throughout the manuscript

Abstract: results section: please include odds ratios for factors associated

Ethics approval: Please have this placed in the right position of the manuscript

Introduction: Please provide a reference for the sentence, “Globally, HIV/AIDS remains a significant public health challenge,…”

State the actual statistics of the proportions of PLHIV made up by adolescents

State the definition of adolescence age group and state reference

What is the burden of malnutrition among adolescents in Ethiopia?

What proportion of adolescents on ART are experiencing comorbidities/virological non-suppression and stigma?

Provide more detail on the selected public health facilities such as location (rural vs urban vs peri-urban), HIV prevalence rates, HIV transmission rates and total clinic enrolments.

What approach was used to triangulate quantitative and qualitative data? Was it a sequential explanatory, sequential exploratory or parallel model?

Were the study tools adapted from previously published tools or developed specifically for the study? If adopted, please provide a reference. If developed specifically for the study, state how they were validated.

What was the rationale for excluding adolescents on ART for less than 3 months?

Please illustrate the sample size calculation.

Provide a sample size allocation table to demonstrate the proportionate allocation of the sample size.

Under section 2.4 Data collection, the content of the first paragraph is best presented under Ethical consideration.

Please clarify whether the healthcare professionals who collected the study data were not involved in routine service provision to the respondents.

Please clarify whether the respondents provided informed consent prior to data collection. Were there instances where assent and/or parental consent were required?

State the brands of weighing scales that were used in the study

Section 2.4.1: please provide references of the literature from which the data tools were adapted.

Provide definitions of the signs of malnutrition that were assessed for from physical assessments

Are viral load assessments part of the baseline assessments at ART initiation?

Under section 2.4.1: Check the second last paragraph and remove repetition of “was assessed”

How is it possible that the bivariate analysis utilized binary logistic regression yet the outcome was not a binary variable?

Were the respondents for the qualitative and quantitative interviews similar?

What was the theoretical basis for the themes that guided qualitative analysis?

Did the team use any software for qualitative analysis?

Section 3.1.3 Clinical Characteristics (HIV-related information): Please re-write, “Half of the participants……” to “More than half of the participants….. before the age of four years.”

In paragraph 2, please correct, “at least one other HIV-positive family members..” to “at least one family member who was HIV positive”

Viral load results are presented for 98.7% of participants, what about the 1.3%?

Please remove bullets in paragraphs in section on mental health

In the discussion section, first paragraph, include a sentence or two on the factors associated with malnutrition.

Comments from Reviewer 3:

3.2.3.

Need to add “of” in the first sentence after “experiences”

In the Discussion paragraph beginning “Gender-based differences”, change interpretation of odds ratio to “male participants had significantly higher odds of being thin and acutely malnourished”

Please submit your revised manuscript by Jun 29 2026 11:59PM. If you will need more time than this to complete your revisions, please reply to this message or contact the journal office at globalpubhealth@plos.org. When you're ready to submit your revision, log on to https://www.editorialmanager.com/pgph/ and select the 'Submissions Needing Revision' folder to locate your manuscript file.

Please include the following items when submitting your revised manuscript:

- A letter that responds to each point raised by the editor and reviewer(s). You should upload this letter as a separate file labeled 'Response to Reviewers'.

- A marked-up copy of your manuscript that highlights changes made to the original version. You should upload this as a separate file labeled 'Revised Manuscript with Track Changes'.

- An unmarked version of your revised paper without tracked changes. You should upload this as a separate file labeled 'Manuscript'.

Guidelines for resubmitting your figure files are available below the reviewer comments at the end of this letter.

As the corresponding author, your ORCID iD is verified in the submission system and will appear in the published article. PLOS supports the use of ORCID, and we encourage all coauthors to register for an ORCID iD and use it as well. Please encourage your coauthors to verify their ORCID iD within the submission system before final acceptance, as unverified ORCID iDs will not appear in the published article. *Only*  the individual author can complete the verification step; PLOS staff *cannot*  verify ORCID iDs on behalf of authors.

We look forward to receiving your revised manuscript.

Kind regards,

Ilse Bloom

Staff Editor

PLOS Global Public Health

**Journal Requirements:**

If the reviewer comments include a recommendation to cite specific previously published works, please review and evaluate these publications to determine whether they are relevant and should be cited. There is no requirement to cite these works unless the editor has indicated otherwise.

**Additional Editor Comments (if provided):**

[Note: HTML markup is below. Please do not edit.]

Reviewers' comments:

[NOTE: If reviewer comments were submitted as an attachment file, they will be attached to this email and accessible via the submission site. Please log into your account, locate the manuscript record, and check for the action link "View Attachments". If this link does not appear, there are no attachment files.]

**Figure Resubmissions:**

While revising your submission, we strongly recommend that you use PLOS’s NAAS tool (https://ngplosjournals.pagemajik.ai/artanalysis) to test your figure files. NAAS can convert your figure files to the TIFF file type and meet basic requirements (such as print size, resolution), or provide you with a report on issues that do not meet our requirements and that NAAS cannot fix.

After uploading your figures to PLOS’s NAAS tool - https://ngplosjournals.pagemajik.ai/artanalysis, NAAS will process the files provided and display the results in the "Uploaded Files" section of the page as the processing is complete. If the uploaded figures meet our requirements (or NAAS is able to fix the files to meet our requirements), the figure will be marked as "fixed" above. If NAAS is unable to fix the files, a red "failed" label will appear above. When NAAS has confirmed that the figure files meet our requirements, please download the file via the download option, and include these NAAS processed figure files when submitting your revised manuscript.

# Author comment: Author response to Decision Letter 4

**DOI:** 10.1371/journal.pgph.0005003.r008

8 May 2026

Attachment Submitted filename: Response to reviewer or Editor.docx

# Editor report: Decision Letter 4

**DOI:** 10.1371/journal.pgph.0005003.r009

**Authors:** Lina Taing, Helen Howard, Andrew Kazibwe, Ilse Bloom, Julia Robinson

1 Jun 2026

Clinical, Lifestyle, Environmental and Dietary Determinants of Malnutrition in Adolescents on Antiretroviral Therapy in Ethiopia

PGPH-D-25-01939R4

Dear Dr. Bore,

We are pleased to inform you that your manuscript 'Clinical, Lifestyle, Environmental and Dietary Determinants of Malnutrition in Adolescents on Antiretroviral Therapy in Ethiopia' has been provisionally accepted for publication in PLOS Global Public Health.

Before your manuscript can be formally accepted you will need to complete some formatting changes, which you will receive in a follow up email. A member of our team will be in touch with a set of requests.

Please note that your manuscript will not be scheduled for publication until you have made the required changes, so a swift response is appreciated.

IMPORTANT: The editorial review process is now complete. PLOS will only permit corrections to spelling, formatting or significant scientific errors from this point onwards. Requests for major changes, or any which affect the scientific understanding of your work, will cause delays to the publication date of your manuscript.

If your institution or institutions have a press office, please notify them about your upcoming paper to help maximize its impact. If they'll be preparing press materials, please inform our press team as soon as possible -- no later than 48 hours after receiving the formal acceptance. Your manuscript will remain under strict press embargo until 2 pm Eastern Time on the date of publication. For more information, please contact globalpubhealth@plos.org.

Thank you again for supporting Open Access publishing; we are looking forward to publishing your work in PLOS Global Public Health.

Best regards,

Julia Robinson

Executive Editor

PLOS Global Public Health

***********************************************************

Reviewer Comments (if any, and for reference):
